# Genetic exploration of the impact of CYP11A1 polymorphisms on the development of PCOS: evidence from a meta-analysis with trial sequential analysis

**DOI:** 10.1038/s41598-026-50710-z

**Published:** 2026-06-03

**Authors:** Arafat Miah, Anamika Datta, Fojla Rabby Shuvo, Md Abdul Barek, Mohammad Safiqul Islam

**Affiliations:** 1 Department of Pharmacy, Noakhali Science and Technology University, Noakhali, 3814 Bangladesh; 2 Laboratory of Pharmacogenomics and Molecular Biology, Department of Pharmacy, Noakhali Science and Technology University, Sonapur, Noakhali, 3814 Bangladesh; 3 Department of Pharmacy, R. P. Shaha University, Narayanganj, 1400 Bangladesh

**Keywords:** CYP11A1, Polymorphism, PCOS, rs4077582, rs11632698, Meta-analysis, Diseases, Endocrinology, Genetics, Medical research

## Abstract

Altering CYP11A1 expression due to polymorphisms can increase the likelihood of developing polycystic ovarian syndrome (PCOS). Several CYP11A1 polymorphisms have been extensively studied for their association with PCOS risk, but the findings have been inconsistent. To clarify this association, a meta-analysis was conducted to evaluate the relationship between various CYP11A1 variants and PCOS. A comprehensive literature search was conducted in PubMed/MEDLINE, Web of Science, the Cochrane Library, ScienceDirect, medRxiv, and Google Scholar, up to August 20, 2025. The quality of each article was evaluated using the Newcastle–Ottawa Scale (NOS). Meta-analysis of genetic association was performed by using the MetaGenyo web-based tool, whereas publication bias was examined by Egger’s test. Additionally, trial sequential analysis (TSA) and in silico gene expression analyses were conducted. Benjamini–Hochberg false discovery rate (FDR) correction was applied to control for multiple testing. In the overall population, we found a statistically significant association between the rs4077582 variant and increased PCOS risk in codominant Model 1 (OR = 2.07), codominant Model 2 (OR = 2.12), dominant Model (OR = 2.10), overdominant Model (OR = 1.54), recessive Model (OR = 1.50), and allelic contrast Model (OR = 1.66). These associations remained significant after FDR correction and trim-and-fill adjustment. For rs11632698, codominant 3 (OR = 1.47) and recessive models (OR = 1.57) were significantly linked with PCOS, although TSA indicates that the current evidence remains inconclusive. Trim-and-fill analysis for rs4077582 models showed that small-study effects slightly attenuated the pooled estimates, but the associations remained statistically significant. In contrast, no significant association was detected in the case of the rs6495096, rs4887139, and rs1484215 polymorphisms. This research suggests that the rs4077582 variant of the CYP11A1 gene may increase the risk of PCOS in the overall population. However, given the observational nature of the included studies and the predefined assumptions of TSA, further research on diverse ethnic groups, including functional and longitudinal studies, is needed to confirm this link more conclusively.

**Supplementary Information:**

The online version contains supplementary material available at 10.1038/s41598-026-50710-z.

## Introduction

Worldwide, 8–20% of women of reproductive age suffer from a complex endocrine disorder known as polycystic ovarian syndrome (PCOS)^1,2^. Primary characteristics include polycystic ovaries, hyperandrogenism, and malfunctions, such as menstrual disruption, infertility, hirsutism, and neonatal problems^3^. The incidence of PCOS ranges from 6 to 26% worldwide, depending on the diagnostic criteria used and the population studied^4–6^. According to a meta-analysis, prevalence rates based on the Rotterdam, NIH, and AE-PCOS Society criteria included 10%, 6%, and 10%, respectively^6^. Among adolescents, the worldwide incidence was 9.8% according to the Rotterdam guidelines and 6.3% according to the International Evidence-based Guideline criteria^6^. A Finnish registry-based study revealed that women with PCOS presented a 47% increased risk of mortality. In comparison to those of controls, the risk of cardiovascular disorders, malignancies, diabetes, and respiratory diseases is notably elevated^7^. However, prevalence rates vary worldwide, with Southeast Asia reporting the highest prevalence (11.4%)^5^. PCOS can begin in the womb and manifest clinically throughout puberty, lasting throughout the reproductive years. Therefore, PCOS may impact women at any time. Even after menopause, women with PCOS have a greater likelihood of developing metabolic diseases, including diabetes, hypertension, and cardiovascular disease^8^.

The pathogenesis involves aberrant gonadotropin secretion, altered ovarian morphology, and impaired insulin action^9^. Risk factors include genetics, lifestyle, environmental contaminants, intestinal dysbiosis, and obesity^10^. Moreover, the pathophysiology of PCOS entails an interaction of hereditary, environmental, and hormonal factors^11^. Hyperandrogenism is a defining biological characteristic of PCOS and is caused principally by excess androgen synthesis by ovarian theca cells^12^. Even under basal conditions, these cells from PCOS ovaries exhibit an increased steroidogenic response, indicating an intrinsic dysregulation of steroid biosynthesis pathways that results in increased androgen production^13^. The CYP11A1 gene is located on chromosome 15q23–q24 and consists of 20 kilobase pairs, comprising nine exons and eight introns. It encodes P450scc, an enzyme that cleaves the side chains of cholesterol, thereby helping to convert cholesterol into pregnenolone. Pregnenolone is the building block for all steroid hormones^14,15^. A study involving 20 affected families revealed a substantial relationship between the CYP11A1 gene and PCOS^14^. Cytochrome P450 enzymes, including CYP11A1, activate procarcinogens and influence estrogen metabolism and transport pathways. Genome-wide association studies (GWASs) revealed several genes that may be linked to PCOS, including FSHB, LHCGR, INSR, CYP11A1, and DENND1A, which create a hierarchical signaling network that influences androgen production^16^.

Genetic predisposition is a crucial aspect of PCOS development, and variations in genes involved in steroidogenic pathways have been extensively studied^17^. CYP11A1 is a crucial gene for steroid hormone synthesis, as it represents the enzyme that facilitates the transformation of cholesterol into pregnenolone, which constitutes the rate-limiting step^18^. Multiple investigations have demonstrated that the level of CYP11A1 expression significantly increases in theca cells of patients with PCOS. This overexpression is caused by both increased promoter activity and increased mRNA stability^13,19^. According to one study, the transcription factor NF1C2 largely mediates the increased baseline and cAMP-stimulated activity of the CYP11A1 promoter in theca cells of PCOS^19^. Furthermore, the mRNA half-life of CYP11A1 in theca cells of individuals with PCOS is significantly prolonged, which is attributed to regulatory elements in the 5′-UTR, hence increasing protein expression and androgen production^19^. However, specific hormonal issues, such as polycystic ovary syndrome, endometrial cancer, prostate cancer, and breast cancer, are caused by genetic variations in the expression of CYP11A1^20,21^. These polymorphisms are believed to play a crucial role in regulating CYP11A1 expression through transcriptional up- or downregulation, which either increases or decreases androgen synthesis^20,21^.

The rs11632698 (A > G), rs6495096 (C > G), and rs1484215 (C > T) are intronic variants, whereas rs4077582 (C > T) and rs4887139 (A > G) are upstream variants of CYP11A1^21,22^. The rs4077582 SNP, located in the 5′ upstream regulatory region of CYP11A1, has garnered attention due to its ability to alter gene expression and mRNA stability, thereby influencing androgen production^22^. Thus, rs4077582 may be linked to changes in androgen levels, specifically testosterone, through LH regulation. LH stimulates the expression of CYP11A1, which is the principal regulator of PCOS in theca cells of growing follicles^22^. On the other hand, variations in the rs11632698 allele are associated with numerous metabolic traits, including obesity, lower blood lipid levels, lower FSH concentrations, altered waist-to-hip ratios, and lower blood glucose levels, suggesting a possible role in metabolic dysregulation associated with PCOS^23^. The GG genotype of rs6495096 is interconnected with an elevated ratio of waist-to-hip and extended infertility, implying that the variant may predispose individuals to hyperandrogenism^21^. Hyperandrogenism is connected to several clinical symptoms, including hirsutism, anovulation, and metabolic abnormalities; hence, rs6495096 is a potential genetic component contributing to the etiology of PCOS. Although the exact molecular mechanism of how rs4887139 affects hyperandrogenism is unknown, a study suggested that this SNP is linked to altered expression of CYP11A1 and is connected to an increased likelihood of acquiring breast cancer^24^. Polymorphisms in the promoter regions of CYP11A1 are linked with high levels of androgen in PCOS. As the SNP rs4887139 is located at the promoter of the CYP11A1 gene, it may modulate transcriptional activity and androgen production, thereby contributing to PCOS^25,26^. Although some studies have examined the genotypic association of rs1484215 with PCOS susceptibility^20,21^, functional studies assessing its transcriptional and translational impact have not yet been explored. However, these polymorphisms may impact CYP11A1 activity, hyperandrogenism, and the development of PCOS.

Several earlier investigations have revealed an association between CYP11A1 gene variants and the risk of PCOS. Case–control experiments have focused on the association of the *CYP11A1* gene rs4077582 polymorphism with the likelihood of PCOS in women^15,22,27–33^. Several studies have suggested that CYP11A1 rs4077582 may contribute to PCOS susceptibility. In the case of rs11632698, a study revealed a significantly increased risk association with PCOS^22^, but others failed to establish a significant association^15,20,27,29^. Similarly, only one study reported that rs6495096 was associated with a substantially lower incidence of PCOS^21^; two more studies, however, failed to find a meaningful association^20,34^. In the case of rs4887139, two studies reported a substantial relationship with PCOS susceptibility^22,27^, whereas one study failed to establish this relationship^20^. However, the recessive model of this study revealed a significant association. Only two studies of rs1484215 failed to establish a significant association between this SNP and PCOS risk^20,21^.

Considering the inconsistent findings from prior investigations, we conducted a meta-analysis of the published data to evaluate these associations. These contradictory results are most likely due to interactions with additional genetic components and environmental influences, as well as variations in detection methods, sample sizes, and experimental designs. Meta-analysis improves the statistical strength and accuracy of effect estimation by combining findings from multiple studies on the same subject^35^. Examining the relationship between CYP11A1 and PCOS may offer crucial insights into the genetic foundations of PCOS conditions, considering its pivotal role in steroidogenesis and potential impact on androgen production. To our knowledge, no meta-analysis has been conducted to date to determine the associations of the rs4077582, rs11632698, rs6495096, rs4887139, and rs1484215 polymorphisms with PCOS, despite several case–control studies having been conducted. Therefore, the present study aimed to conduct a comprehensive meta-analysis of the association between these five SNPs and PCOS risk within a unified analytical framework. Additionally, this study applies trial sequential analysis (TSA) to assess the conclusiveness of evidence and integrates in silico expression analyses to explore potential functional relevance. Collectively, these approaches provide a more robust, multidimensional understanding of CYP11A1’s genetic contribution to PCOS susceptibility. Moreover, this present study advances existing evidence by combining data from multiple populations to provide a more precise pooled estimate of the association between these polymorphisms and PCOS susceptibility in the global population.

## Methods

### Literature search strategy

This investigation was registered in PROSPERO (CRD420251175314) and reported in compliance with the PRISMA 2020 recommendations (Supplementary file 1)^36^. Data were obtained from multiple databases using predefined search terms. The most relevant searches included “CYP11A1”, "rs4077582 and PCOS", "rs11632698 and PCOS", "rs6495096 and PCOS", "rs4887139 and PCOS", "rs1484215 and PCOS", "CYP11A1 variants and PCOS risk", "CYP11A1 and PCOS susceptibility", "linkage/correlation/association/impact/relationship", "CYP11A1 polymorphism and PCOS in different ethnic populations", "case‒control studies", and "prognosis/development". These terms were searched in the following bibliographic databases: PubMed/MEDLINE, Web of Science, Scopus, the Cochrane Library (CENTRAL), ScienceDirect, medRxiv, and Google Scholar from inception to August 20, 2025. Publisher platforms (e.g., Wiley, Springer, BMC) were accessed through these databases where applicable. This investigation focused on any language literature examining the relationship between PCOS risk and CYP11A1 gene polymorphisms (rs4077582, rs11632698, rs6495096, rs4887139, and rs1484215). Furthermore, data were retrieved from papers published until August 20, 2025. We conducted a thorough analysis of the citations and references in the chosen papers and published reviews to identify missing publications. The detailed PubMed and Web of Science search string, including Boolean operators and filters, is provided in Table S3.

Database-specific search strategies were developed for each platform using combinations of controlled vocabulary (e.g., MeSH terms in PubMed) and free-text keywords. Boolean operators (“AND”, “OR”), truncation, and field tags were applied where appropriate. No language restrictions were imposed. The complete electronic search strategies for PubMed, Web of Science, and Scopus are provided in Supplementary Table S3 to ensure reproducibility.

### Eligibility criteria

For the purpose of determining eligibility, certain inclusion and exclusion criteria were used. The following criteria were used to choose papers for this study: (a) case–control investigation, (b) examination of the association between rs4077582, rs11632698, rs6495096, rs4887139, and rs1484215 with PCOS, (c) genotypic information for the control group and patients, (d) investigations using any validated human DNA source. The exclusion criteria were as follows: (a) not designed as case‒control studies, (b) studies with insufficient genotypic data for odds ratio (OR) calculations, (c) studies on animals, and (d) letters to the editors, reviews, or commentaries.

### Data extraction and quality assessment

Data were extracted from the selected publications and then entered into a Microsoft Excel spreadsheet that had been previously created. Genotype data and relevant study characteristics were independently extracted by two reviewers **(AM and FRS)**. Any discrepancies were resolved through discussion and consensus, and, when necessary, by consultation with a third reviewer **(MSI)**. All the authors used this spreadsheet to extract data independently. Data collection focused on fundamental features of the included papers, such as year of publication, first author, ethnicity, nation, source of control, sample size, genotype distribution, and genotypic approach. The specifications of the Newcastle–Ottawa Scale (NOS) were employed to evaluate the reliability of the chosen observational case–control research designs^37^. There are three categories for quality: poor (less than four), medium (four to six), and excellent (score of seven or above). Three key attributes were assessed: research results, comparability, and selection criteria.

### Statistical analysis

We analyzed data from publications using MetaGenyo, a web-based tool for meta-analysis of gene-related association studies (https://metagenyo.genyo.es/)^38^. The associations of risk were quantified as odds ratios (ORs) accompanied by 95% confidence intervals (CIs). The association between genetic polymorphisms and the probability of PCOS was evaluated via seven distinctive genotypic models: codominant model 1 (COD1), codominant model 2 (COD2), codominant model 3 (COD3), dominant model (DM), overdominant model (OM), recessive model (RM), and allelic model (AM). Evaluating multiple models allows identification of both additive and non-additive effects and helps capture diverse inheritance patterns that may influence PCOS susceptibility across different populations. *p*-value less than 0.05 was considered statistically significant. The I^2^ statistic was used to assess study heterogeneity, and heterogeneity was considered substantial if I^2^ ≥ 50 or *P*_H_ < 0.10. The random-effects model was used in scenarios where the heterogeneity value was larger than or equal to 50% or where the *P*_H_ was < 0.1. In contrast, a fixed effects model was used for nonsignificant heterogeneity. Publication bias was assessed using Egger’s test, with p-values greater than 0.05 indicating the absence of publication bias^39^. Additionally, we visually assessed the presence of publication bias through the generation of funnel plots. A sensitivity analysis was conducted in this investigation for each SNP to assess the credibility of the findings by sequentially eliminating individual research. In addition, subgroup analyses based on genotyping methods (ethnicity and genotyping methods) were used to investigate the impact of CYP11A1 rs4077582 on PCOS patients. To evaluate the influence of studies that deviated from HWE in control groups, a sensitivity analysis for rs4077582 was performed after excluding six studies^15,22,28,30,32,and33]^.

Because five SNPs were evaluated under seven genetic models (35 total comparisons), the potential inflation of Type I error was considered. The allelic (additive) model was pre-specified as the primary genetic model, whereas the remaining models were considered secondary exploratory analyses. To control for multiple testing, Benjamini–Hochberg false discovery rate (FDR) correction was applied to overall meta-analysis p-values for SNPs showing nominal statistical significance. FDR-adjusted q-values < 0.05 were considered statistically significant.

### Credibility and certainty assessment

To evaluate the cumulative credibility of significant associations, the Venice criteria framework for genetic association studies was applied. This approach grades evidence based on three domains: (1) amount of evidence (sample size and statistical power), (2) replication consistency (heterogeneity and consistency across studies), and (3) protection from bias (publication bias, small-study effects, and multiple testing correction). Each domain was graded as strong (A), moderate (B), or weak (C). The overall credibility of the evidence was assessed based on the combined grading.

### Trial sequential analysis

To mitigate random error, we implemented trial sequential analysis (TSA) and determined if the sample size was sufficient to provide significant results for rs4077582, rs11632698, rs6495096, rs4887139, and rs1484215 from the perspective of AM. TSA was implemented with a 5% overall type I error, 80% statistical test power, 20% relative risk reduction, and a 95% confidence interval. The trial sequential monitoring limits and necessary information size (RIS) were then established. If the cumulative Z-curve crosses the trial sequential monitoring boundary or the accrued sample size reaches the required information size (RIS), the evidence may be considered statistically stable under the predefined assumptions; however, this does not preclude the need for additional validation studies. The TSA 0.9.5.10 beta software was implemented^40^.

### In silico analysis of gene expression

We estimated the impact of the rs4077582, rs11632698, rs6495096, rs4887139, and rs1484215 SNPs on CYP11A1 gene expression in ovarian tissues via expression quantitative trait locus (eQTL) analysis via the GTEx portal website (http://www.gtexportal.org/home/). To visualize gene expression, we generated violin plots^41^.

## Results

### Studies included in the meta‑analysis

**Fig. 1:**
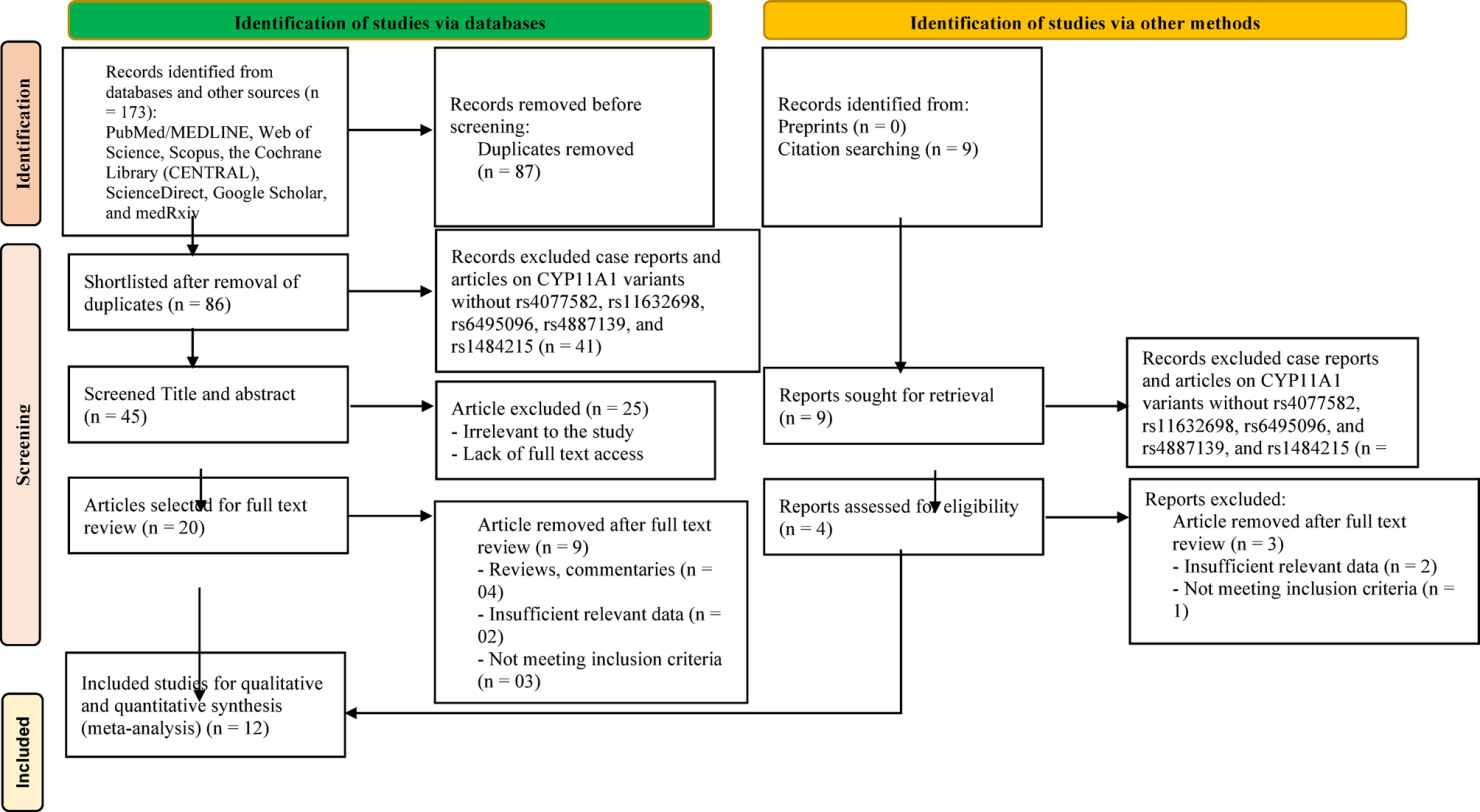
PRISMA flow chart for the assessment of selected studies for the meta-analysis.

Figure 1 illustrates the search procedure and selection based on the PRISMA 2020 recommendations. A complete literature search revealed 173 records from databases such as PubMed, BMC, Wiley, Springer, Web of Science, the Cochrane Library, Google Scholar, and Science Direct. After 87 duplicates were deleted, 86 records were saved for screening. A total of 45 records were further examined via title and abstract screening, with 20 publications selected for full-text reviews. Nine papers were omitted because they were reviews/commentaries (four), lacked adequate relevant data (two), and did not match the inclusion criteria (three). In addition to database searches, 9 additional records were observed via various approaches, including preprints (n = 0) and citation searches (n = 9). Following the retrieval and eligibility assessment of four papers, three were eliminated due to inadequate data (n = 2) and non-eligibility (n = 1), but there was one more paper added to the meta-analysis. As a consequence, the final meta-analysis included 12 qualifying papers^15,20–22,27–34^.

We analyzed data from 12 studies comprising 2267 cases and 2262 controls for genotyping and association analyses between CYP11A1 SNPs (rs4077582, rs11632698, rs6495096, rs4887139, and rs1484215) and PCOS; however, the number of studies contributing data varied by SNP depending on availability in the primary publications. Among the 12 studies, nine studies with 1,857 cases and 1,838 controls considered rs4077582, five studies reported 1,448 cases and 1,447 controls for rs11632698, three studies included 410 cases and 422 controls for rs6495096, three studies included 845 cases and 913 controls for rs4887139, and two studies included 385 cases and 399 controls for rs1484215. Nine of the studies were conducted in Asian populations, whereas three were conducted in Caucasian populations. In the context of genotyping methods, many studies have utilized either PCR–RFLP or ARMS-PCR for variant detection. While the twelve studies recruited HB controls, only one study recruited PB controls. Good quality was indicated by the majority of included studies, which received an NOS quality assessment score of ≥ 6 (Supplementary file 2). The distinguishing features of the chosen studies, along with their genotype frequencies, p-values for HWE, and NOS scores, are presented in Table 1.

**Table 1:** Genotypic and characteristic information of the selected studies for meta-analysis to detect the association *CYP11A1* rs4077582, rs11632698, rs6495096, rs4887139, and rs1484215 with PCOS.

**Study ID**	**Year**	**Country**	**Ethnicity**	**Genotyping Method**	**Controls Source**	**Cases **	**Controls**	**Genotypes**						**HWE** ***p*-value**	**NOS score**
								**Cases**			**Controls**				
*CYP11A1* rs4077582															
								TT	CT	CC	TT	CT	CC		
Gao et al.^27^	2010	China	Asian	PCR-RFLP	HB	290	344	72	144	74	58	167	119	0.9637	7
Zhang et al. ^15^	2012	China	Asian	PCR-RFLP	HB	314	314	25	221	68	29	180	105	0.0001	5
Abdel-mageed et al. ^28^	2016	Egypt	Caucasian	PCR-RFLP	HB	52	52	3	20	29	2	6	44	0.0153	6
Chen et al. ^29^	2016	China	Asian	PCR-RFLP	HB	289	220	73	144	72	42	102	76	0.458	7
Kaur et al. ^22^	2021	India	Asian	PCR-RFLP	HB	270	270	62	111	97	38	90	142	0.0004	8
Sajid et al. ^30^	2024	Pakistan	Asian	ARMS-PCR	HB	150	150	59	49	42	48	33	69	0.00	7
Aboelros et al. ^31^	2025	Egypt	Caucasian	TaqMan	HB	50	50	14	22	14	4	18	28	0.649	7
Wazir et al. ^32^	2025	Pakistan	Asian	Sanger-sequencing	PB	142	138	36	74	32	20	46	72	0.0088	7
Bashir et al. ^33^	2025	Pakistan	Asian	ARMS-PCR	HB	300	300	118	98	84	96	66	138	0.00	6
Total						1857	1838	462	883	512	337	708	793		

*CYP11A1* rs11632698															
								GG	AG	AA	GG	AG	AA		
Gao et al. ^27^	2010	China	Asian	PCR-RFLP	HB	290	344	106	130	54	94	188	62	0.0571	7
Zhang et al. ^15^	2012	China	Asian	PCR-RFLP	HB	314	314	10	150	154	11	145	158	0.0012	5
Chen et al. ^29^	2016	China	Asian	PCR-RFLP	HB	289	220	18	112	159	9	73	138	0.8661	7
Shan et al. ^20^	2016	China	Asian	MassARRAY iPLEX	HB	285	299	4	66	215	6	83	210	0.5034	8
Kaur et al. ^22^	2021	India	Asian	PCR-RFLP	HB	270	270	46	98	126	21	75	174	0.0028	8
Total						1448	1447	184	556	708	141	564	742		

*CYP11A1 rs*6495096															
								GG	CG	CC	GG	CG	CC		
Shan et al. ^20^	2016	China	Asian	MassARRAY iPLEX	HB	285	297	48	126	111	34	144	119	0.3327	8
Fathy et al. ^21^	2023	Iran	Caucasian	T-ARMS-PCR	HB	100	100	2	82	16	2	62	36	0.0001	7
Rao et al. ^34^	2025	Pakistan	Asian	AS-PCR	HB	25	25	2	16	7	5	12	8	0.8967	4
Total						410	422	52	224	134	41	218	163		

*CYP11A1 *rs4887139															
								GG	AG	AA	GG	AG	AA		
Gao et al. ^27^	2010	China	Asian	PCR-RFLP	HB	290	344	3	50	237	8	75	261	0.3519	7
Shan et al. ^20^	2016	China	Asian	MassARRAY iPLEX	HB	285	299	36	26	222	23	36	240	0.00	8
Kaur et al. ^22^	2021	India	Asian	PCR-RFLP	HB	270	270	207	58	5	239	30	1	0.9549	8
Total						845	913	246	134	464	270	141	502		

*CYP11A1 *rs1484215															
								TT	CT	CC	TT	CT	CC		
Shan et al. ^20^	2016	China	Asian	MassARRAY iPLEX	HB	285	299	29	117	139	24	126	149	0.7125	8
Fathy et al. ^21^	2023	Iran	Caucasian	T-ARMS-PCR	HB	100	100	11	75	14	21	63	16	0.0085	7
Total						385	399	40	192	153	45	189	165		

### Association of the CYP11A1 rs4077582 polymorphism with PCOS

**Fig. 2:**
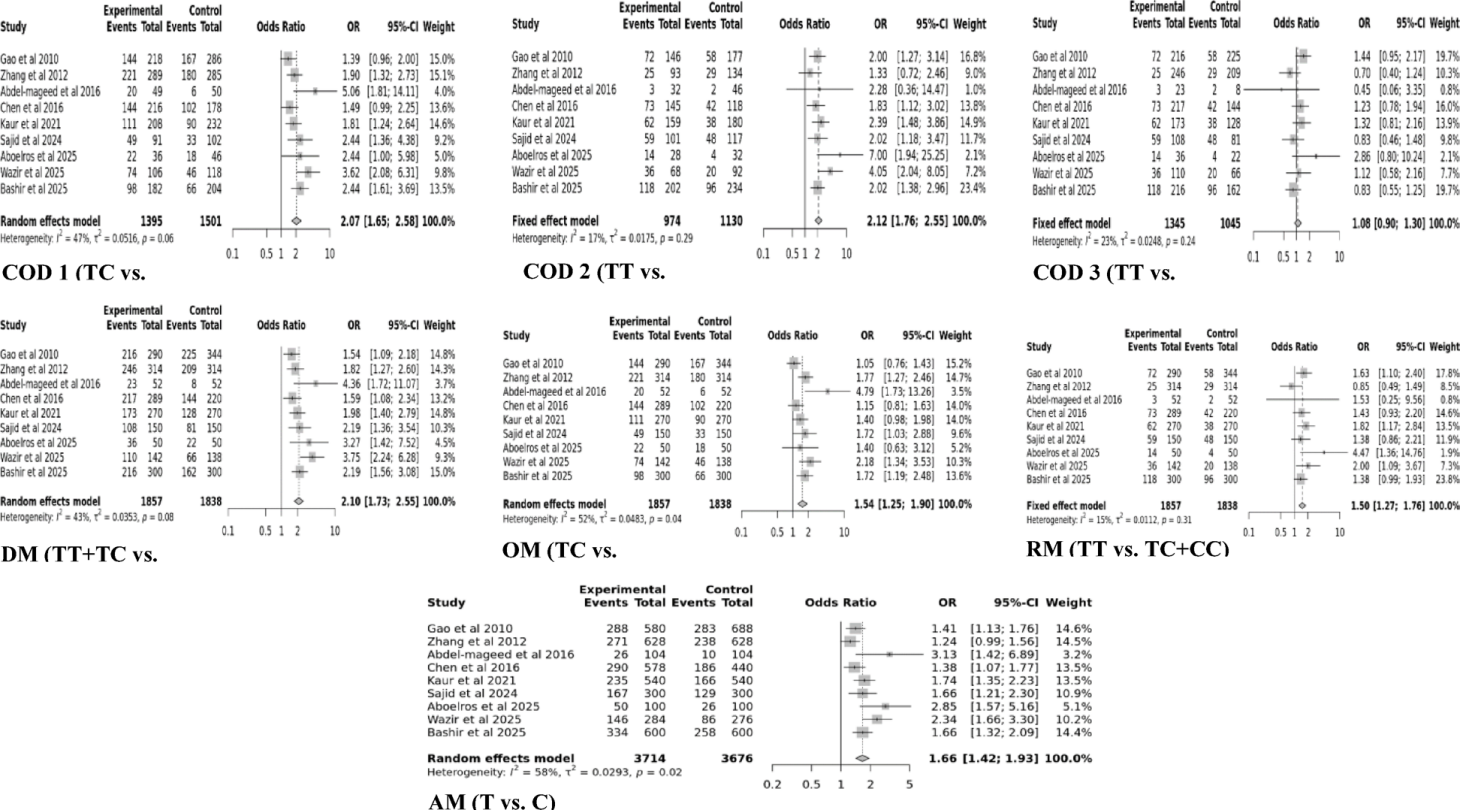
Forest plot showing the association of rs4077582 polymorphism and PCOS under different genetic models.

The meta-analysis comprised nine studies to examine the association between the CYP11A1 rs4077582 polymorphism and the risk for PCOS, and the meta-analysis findings are described in Table 2. Statistically significant relationships were found across different genetic models in the overall analysis. Elevated PCOS risk was identified within COD1 (TC vs. CC: OR = 2.07, 95% CI = 1.65–2.58, p < 0.001), COD2 (TT vs. CC: OR = 2.12, 95% CI = 1.76–2.55, p < 0.001), DM (TT + TC vs. CC: OR = 2.10, 95% CI = 1.73–2.55, p < 0.001), OM (TC vs. TT + CC: OR = 1.54, 95% CI = 1.25–1.90, p < 0.001), RM (TT vs. TC + CC: OR = 1.50, 95% CI = 1.27–1.76, p < 0.001), and AM (T vs. C: OR = 1.66, 95% CI = 1.42–1.93, p < 0.001). However, COD3 (TT vs. TC) did not have a statistically significant association (OR = 1.08, 95% CI = 0.90–1.30, p = 0.417). Figure 2 shows forest plots for various genetic models associated with rs4077582.

**Table 2:** Meta-analysis of the link between *CYP11A1* rs4077582 polymorphism with PCOS.

Model	Genotyping method	Number of studies	Test of association			Test of heterogeneity			Publication bias
			OR	95% CI	P_Z_	Model	P_H_	I^2^ (%)	p-val (Egger’s test)
COD1 (TC vs. CC)	Overall	9	2.07	1.65–2.58	**0.000**	Random	0.056	47.27	0.039
	Asian	7	1.96	1.57–2.45	**0.000**	Random	0.073	47.98	0.068
	Caucasian	2	3.35	1.70–6.57	**0.000**	Fixed	0.295	8.81	NA
	PCR–RFLP	5	1.70	1.41–2.05	**0.000**	Fixed	0.173	37.21	0.099
	ARMS-PCR	2	2.44	1.74–3.42	**0.000**	Fixed	1.000	0.00	NA
COD2 (TT vs. CC)	Overall	9	2.12	1.76–2.55	**0.000**	Fixed	0.290	17.19	0.229
	Asian	7	2.07	1.71–2.49	**0.000**	Fixed	0.395	4.10	0.604
	Caucasian	2	4.86	1.69–13.95	**0.003**	Fixed	0.328	0.00	NA
	PCR–RFLP	5	1.93	1.50–2.47	**0.000**	Fixed	0.690	0.00	0.837
	ARMS-PCR	2	2.02	1.48–2.76	**0.000**	Fixed	1.000	0.00	NA
COD3 (TT vs. TC)	Overall	9	1.08	0.90–1.30	0.417	Fixed	0.236	23.31	0.936
	Asian	7	1.06	0.88–1.28	0.509	Fixed	0.283	19.21	0.466
	Caucasian	2	1.68	0.57–4.94	0.342	Fixed	0.127	57.01	NA
	PCR–RFLP	5	1.18	0.93–1.49	0.164	Fixed	0.266	23.25	0.249
	ARMS-PCR	2	0.83	0.59–1.16	0.271	Fixed	1.000	0.00	NA
DM (TT + TC vs. CC)	Overall	9	2.10	1.73–2.55	**0.000**	Random	0.084	42.55	0.026
	Asian	7	1.96	1.70–2.26	**0.000**	Fixed	0.132	38.96	0.104
	Caucasian	2	3.72	2.00–6.92	**0.000**	Fixed	0.652	0.00	NA
	PCR–RFLP	5	1.79	1.50–2.13	**0.000**	Fixed	0.296	18.6	0.084
	ARMS-PCR	2	2.19	1.66–2.89	**0.000**	Fixed	1.000	0.00	NA
OM (TC vs. TT + CC)	Overall	9	1.54	1.25–1.90	**0.000**	Random	0.035	51.65	0.092
	Asian	7	1.48	1.22–1.80	**0.000**	Random	0.079	46.98	0.158
	Caucasian	2	2.48	0.74 -8.29	0.139	Random	0.062	71.20	NA
	PCR–RFLP	5	1.44	1.06–1.95	**0.020**	Random	0.018	66.53	0.161
	ARMS-PCR	2	1.72	1.28–2.32	**0.000**	Fixed	1.000	0.00	NA
RM (TT vs. TC + CC)	Overall	9	1.50	1.27–1.76	**0.000**	Fixed	0.313	14.56	0.402
	Asian	7	1.46	1.24–1.73	**0.000**	Fixed	0.416	1.04	0.868
	Caucasian	2	3.25	1.20–8.83	**0.020**	Fixed	0.337	0.00	NA
	PCR–RFLP	5	1.46	1.17–1.82	**0.001**	Fixed	0.303	17.47	0.693
	ARMS-PCR	2	1.38	1.05–1.81	**0.022**	Fixed	1.000	0.00	NA
AM (T vs. C)	Overall	9	1.66	1.42–1.93	**0.000**	Random	0.015	57.77	0.013
	Asian	7	1.56	1.36–1.80	**0.000**	Random	0.062	49.95	0.074
	Caucasian	2	2.95	1.83–4.74	**0.000**	Fixed	0.849	0.00	NA
	PCR–RFLP	5	1.44	1.29–1.62	**0.000**	Fixed	0.105	47.79	0.135
	ARMS-PCR	2	1.66	1.38–2.01	**0.000**	Fixed	1.000	0.00	NA

*Bold values indicate statistically significant (*p* < 0.05).

To assess the association between polymorphisms and the risk of PCOS in Asian and Caucasian populations, subgroup analyses were conducted (Table 2). Significant relationships were consistently found across most genetic models in the Asian subgroup (Fig. 7). In the codominant models, the risk was 1.96 times greater for TC genotype carriers than for CC carriers (OR = 1.96, 95% CI 1.57–2.45, p < 0.001) and 2.07 times greater for TT carriers than for CC carriers (OR = 2.07, 95% CI 1.71–2.49, p < 0.001). A significant association was also found in the DM (TT + TC vs. CC) (OR = 1.96, 95% CI 1.70–2.26, p < 0.001). Additionally, Asian populations showed increased susceptibility to PCOS in the OM (OR = 1.48, 95% CI = 1.22–1.80, p < 0.001), RM (TT vs. TC + CC; OR = 1.46, 95% CI 1.24–1.73, p < 0.001), and AM (T vs. C; OR = 1.56, 95% CI 1.36–1.80, p < 0.001). However, on the basis of only two studies, the Caucasian subgroup also presented significant associations (Fig. 8). The risk of PCOS was 4.86 times greater for TT carriers than for CC carriers (OR = 4.86, 95% CI 1.69–13.95, p = 0.003) and more than three times greater for carriers of TC genotype (OR = 3.35, 95% CI 1.71–6.57, p < 0.001). Similarly, among Caucasians, the DM (OR = 3.72, 95% CI 2.00–6.92, p < 0.001), RM (OR = 3.25, 95% CI 1.20–8.83, p = 0.020), and AM (OR = 2.95, 95% CI 1.83–4.74, p < 0.001) were significantly related to PCOS risk.

Subgroup analysis using the genotyping method revealed consistent relationships across the ARMS-PCR and PCR–RFLP methodologies (Table 2). The PCR–RFLP subgroup (n = 5) showed significant associations under all the models except COD3, with the most pronounced associations observed in the COD1 (OR = 1.70, 95% CI = 1.41–2.05, p < 0.001), COD2 (OR = 1.93, 95% CI = 1.50–2.47, p < 0.001), and DM (OR = 1.79, 95% CI = 1.50–2.13, p < 0.001). Similarly, the OM (OR = 1.44, 95% CI = 1.06–1.95, p = 0.001), RM (OR = 1.46, 95% CI = 1.17–1.82, p = 0.016), and AM (OR = 1.44, 95% CI = 1.29–1.62, p = 0.001) also revealed significant association (Fig. 9). In the ARMS-PCR-based studies (n = 2), all the models except the codominant Model 3 reported statistically significant associations with higher PCOS risk within the COD1 (OR = 2.44, 95% CI = 1.74–3.42, p < 0.001) and the DM (OR = 2.19, 95% CI = 1.66–2.89, p < 0.001). Increased PCOS risk was also found in COD2 (OR = 2.02, 95% CI = 1.48–2.76, p < 0.001), OM (OR = 1.72, 95% CI = 1.28–2.32, p < 0.001), RM (OR = 1.38, 95% CI = 1.05–1.81, p = 0.003), and AM (OR = 1.66, 95% CI = 1.38–2.01, p < 0.001) (Fig. 10).

### Association of the CYP11A1 rs11632698 polymorphism with PCOS

**Fig. 3:**
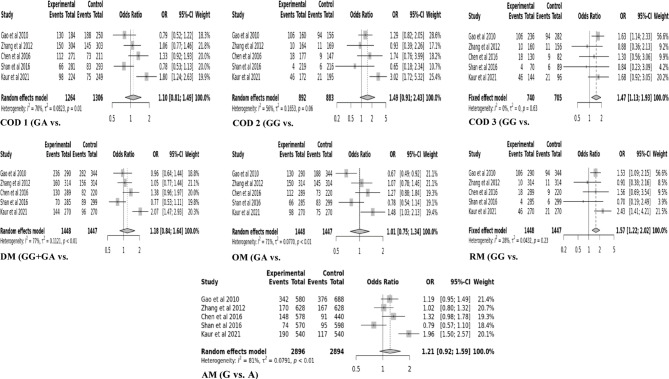
Forest plot showing the association of rs11632698 polymorphism and PCOS under different genetic models.

The meta-analysis included five studies that evaluated the association between the CYP11A1 rs11632698 polymorphism and PCOS susceptibility, and detailed outcomes are provided in Table 3** and **Fig. 3. Across all the genetic models, COD3 (GG vs. GA: OR = 1.47, 95% CI = 1.13–1.93, p = 0.005) and RM (GG vs. GA + AA: OR = 1.57, 95% CI = 1.22–2.02, p < 0.001) were significantly related. However, TSA indicated that the required information size was not reached, suggesting that these findings remain preliminary and should be interpreted with caution. No significant association was found in COD1 (GA vs. AA: OR = 1.14, 95% CI = 0.72–1.80, p = 0.577) and COD2 (GG vs. AA: OR = 1.36, 95% CI = 0.50–3.69, p = 0.545). Similarly, DM (GG + GA vs. AA: OR = 1.19, 95% CI = 0.68–2.07, p = 0.542), OM (GA vs. GG + AA: OR = 1.08, 95% CI = 0.76–1.51, p = 0.675), and AM (G vs. A: OR = 1.17, 95% CI = 0.70–1.97, p = 0.548) could not suggest any substantial association.

**Table 3:** Meta-analysis of the link between *CYP11A1* rs11632698 polymorphism with PCOS.

Model	Subgroup	Number of studies	Test of association			Test of heterogeneity			Publication bias
			OR	95% CI	P_Z_	Model	P_H_	I^2^ (%)	p-val (Egger’s test)
COD1 (GA vs. AA)	Overall	5	1.10	0.81—1.49	0.537	Random	0.011	69.64	0.791
COD2 (GG vs. AA)	Overall	5	1.49	0.91—2.44	0.113	Random	0.060	55.85	0.495
COD3 (GG vs. GA)	Overall	5	1.47	1.13—1.93	**0.005**	Fixed	0.628	0.00	0.072
DM (GG + GA vs. AA)	Overall	5	1.18	0.84—1.64	0.340	Random	0.001	77.38	0.716
OM (GA vs. GG + AA)	Overall	5	1.00	0.75—1.34	0.962	Random	0.007	71.39	0.464
RM (GG vs. GA + AA)	Overall	5	1.57	1.22—2.02	**0.000**	Fixed	0.232	28.46	0.419
AM (G vs. A)	Overall	5	1.21	0.92—1.59	0.179	Random	0.000	80.77	0.762

*Bold values indicate statistically significant (*p* < 0.05).

### Association of the CYP11A1 rs6495096 polymorphism with PCOS

**Fig. 4:**
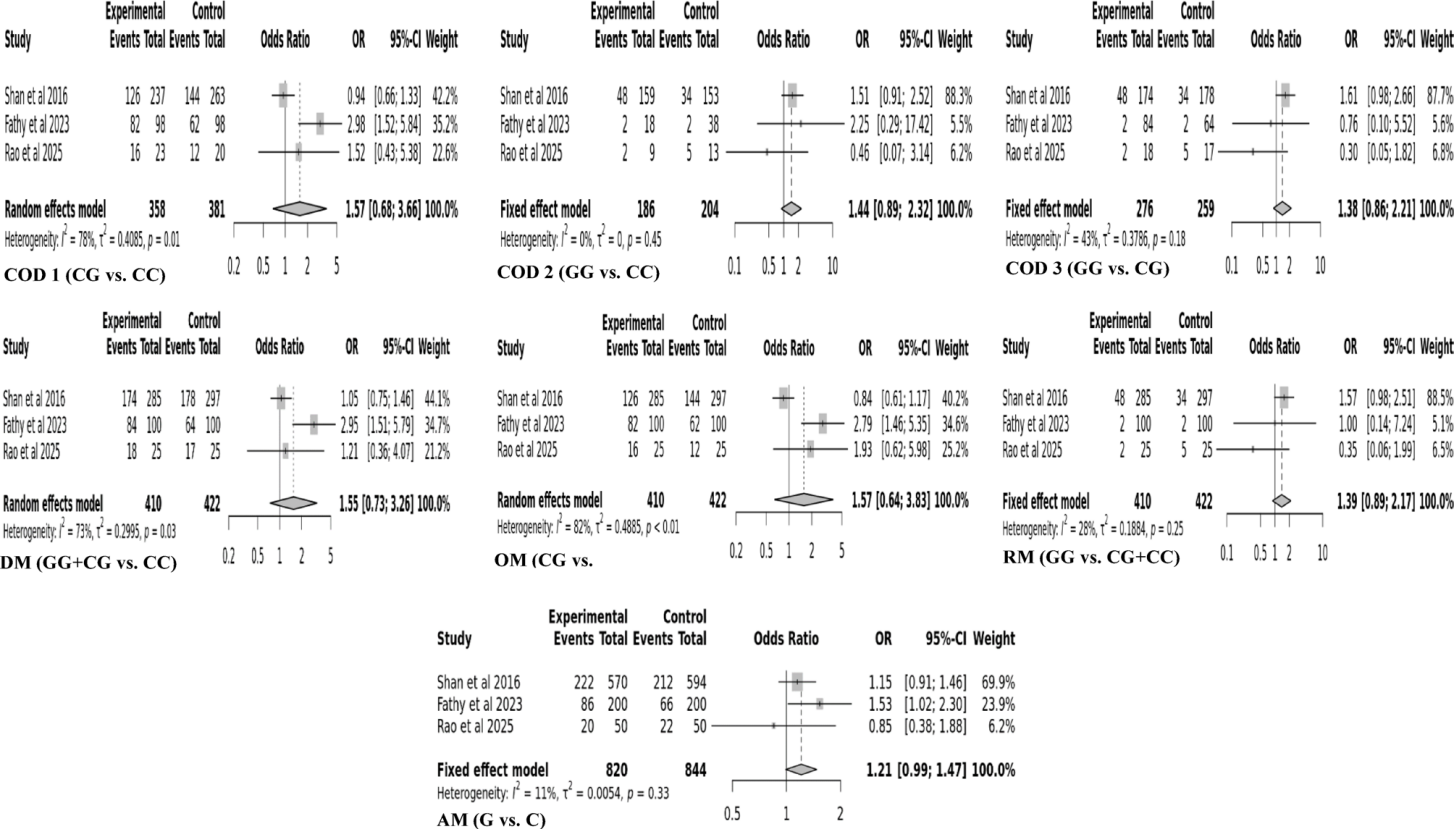
Forest plot showing the association of rs6495096 polymorphism and PCOS under different genetic models.

Three investigations were undertaken to determine the association between the *CYP11A1* rs6495096 polymorphism and PCOS vulnerability, as described in Table 4 and Fig. 4. There was no statistically significant association found in any of the seven genetic models. In the codominant models, no significant association was detected between CG and CC (OR = 1.57, 95% CI = 0.68–3.66, p = 0.294), GG and CC (OR = 1.44, 95% CI = 0.89–2.32, p = 0.138), or GG and CG (OR = 1.38, 95% CI = 0.86–2.21, p = 0.177). Similarly, the DM (GG + CG vs. CC: OR = 1.55, 95% CI = 0.73–3.26, p = 0.251), OM (CG vs. GG + CC: OR = 1.57, 95% CI = 0.64–3.83, p = 0.321), and RM (GG vs. CG + CC: OR = 1.39, 95% CI = 0.89–2.17, p = 0.148) revealed no significant relationships. The AM also revealed a borderline but nonsignificant association (G vs. C: OR = 1.21, 95% CI = 0.99–1.47, p = 0.062).

**Table 4:** Meta-analysis of the link between *CYP11A1* rs6495096 polymorphism with PCOS.

Model	Subgroup	Number of studies	Test of association			Test of heterogeneity			Publication bias
			OR	95% CI	P_Z_	Model	P_H_	I^2^ (%)	p-val (Egger’s test)
COD1 (CG vs. CC)	Overall	3	1.57	0.68—3.66	0.294	Radom	0.011	77.68	0.554
COD2 (GG vs. CC)	Overall	3	1.44	0.89—2.32	0.138	Fixed	0.454	0.00	0.717
COD3 (GG vs. CG)	Overall	3	1.38	0.86—2.21	0.177	Fixed	0.176	42.54	0.280
DM (GG + CG vs. CC)	Overall	3	1.55	0.73—3.26	0.251	Random	0.026	72.72	0.635
OM (CG vs. GG + CC)	Overall	3	1.57	0.64—3.83	0.321	Random	0.003	82.44	0.422
RM (GG vs. CG + CC)	Overall	3	1.39	0.89—2.17	0.148	Fixed	0.250	27.81	0.369
AM (G vs. C)	Overall	3	1.21	0.99—1.47	0.062	Fixed	0.327	10.56	0.920

### Association of the CYP11A1 rs4887139 polymorphism with PCOS

**Fig. 5:**
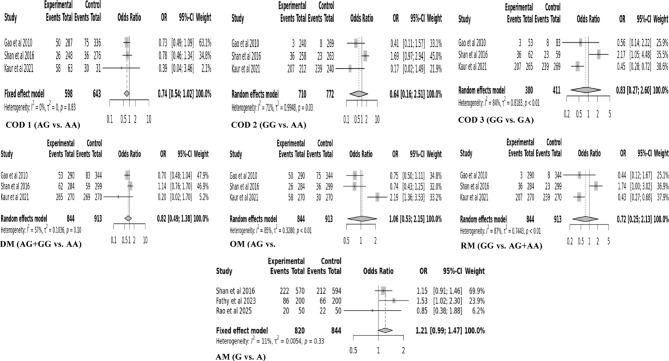
Forest plot showing the association of rs4887139 polymorphism and PCOS under different genetic models.

For this polymorphism, the overall meta-analysis included three studies. In all the evaluated genetic models, no statistically noteworthy association between the variation and PCOS likelihood was found, as presented in Table 5 and Fig. 5. The results of the codominant comparisons were not statistically significant (AG vs. AA: OR = 0.74, 95% CI 0.54–1.02, p = 0.062; GG vs. AA: OR = 0.64, 95% CI 0.16–2.51, p = 0.526; GG vs. AG: OR = 0.83, 95% CI 0.27–2.60, p = 0.751). The OM (AG vs. GG + AA: OR = 1.06, 95% CI 0.53–2.15, p = 0.866), RM (GG vs. AG + AA: OR = 0.72, 95% CI 0.25–2.13, p = 0.555), DM (AG + GG vs. AA: OR = 0.82, 95% CI 0.49–1.38, p = 0.460), and AM (G vs. A: OR = 0.75, 95% CI 0.40–1.39, p = 0.358) also did not show significant associations.

**Table 5:** Meta-analysis of the link between *CYP11A1* rs4887139 polymorphism with PCOS.

Model	Subgroup	Number of studies	Test of association			Test of heterogeneity			Publication bias
			OR	95% CI	P_Z_	Model	P_H_	I^2^ (%)	p-val (Egger’s test)
COD1 (AG vs. AA)	Overall	3	0.74	0.54—1.02	0.062	Fixed	0.828	0.00	0.353
COD2 (GG vs. AA)	Overall	3	0.64	0.16—2.51	0.526	Random	0.030	71.45	0.080
COD3 (GG vs. GA)	Overall	3	0.83	0.27—2.60	0.751	Random	0.002	84.28	0.767
DM (AG + GG vs. AA)	Overall	3	0.82	0.49—1.38	0.460	Random	0.096	57.38	0.588
OM (AG vs. GG + AA)	Overall	3	1.06	0.53—2.15	0.866	Random	0.001	85.27	0.839
RM (GG vs. AG + AA)	Overall	3	0.72	0.25—2.13	0.555	Random	0.000	86.84	0.988
AM (G vs. A)	Overall	3	0.75	0.40—1.39	0.358	Random	0.000	88.24	0.220

### Association of the CYP11A1 rs1484215 polymorphism with PCOS

**Fig. 6:**
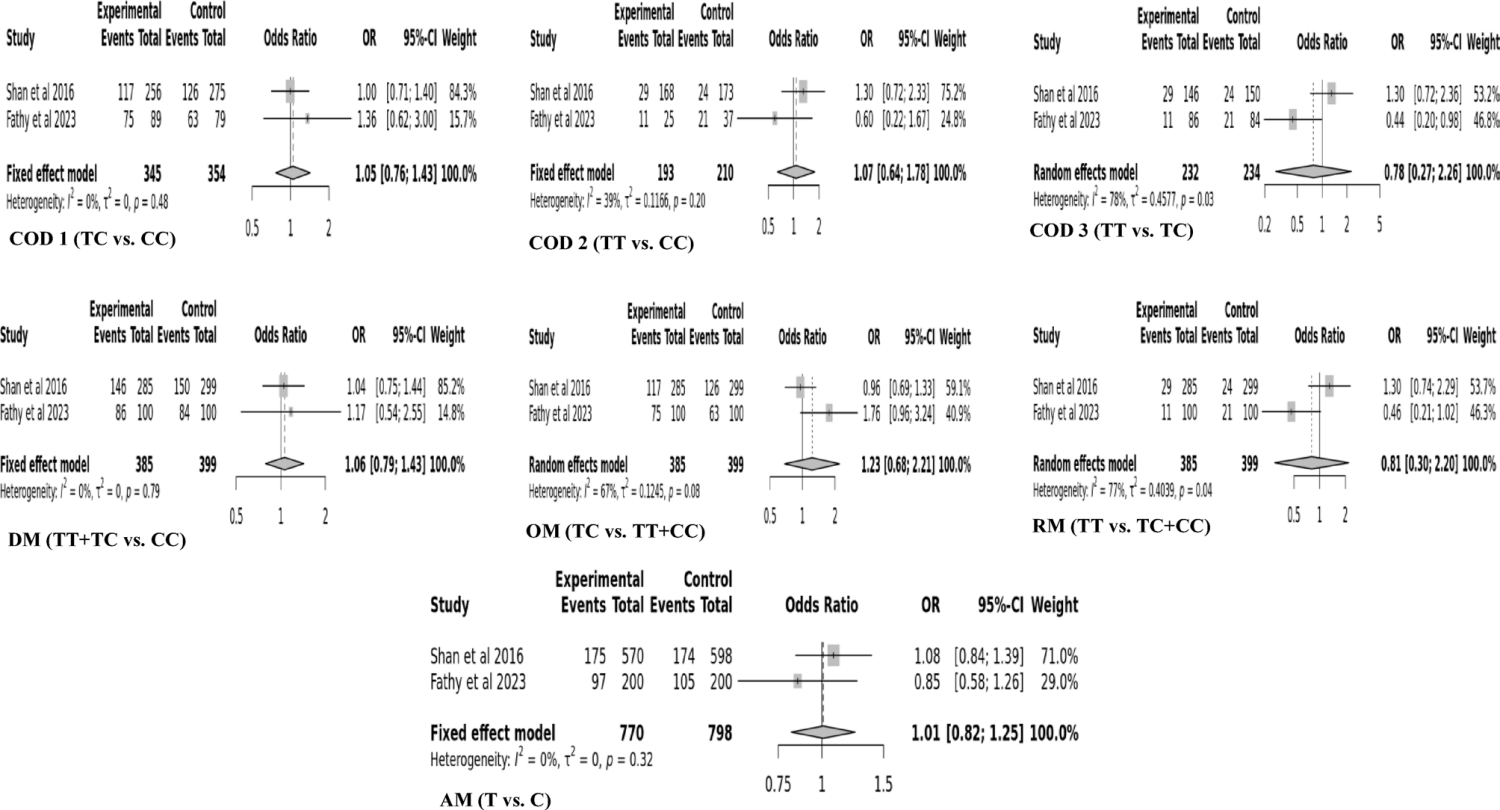
Forest plot showing the association of rs1484215 polymorphism and PCOS under different genetic models.

Table 6 and Fig. 6 demonstrate that this polymorphism did not significantly associate with PCOS in any genetic model. TT vs. CC (OR = 1.07, 95% CI 0.64–1.78, p = 0.797), CT vs. CC (OR = 1.05, 95% CI 0.76–1.43, p = 0.781), and TT vs. CT (OR = 0.78, 95% CI 0.27–2.26, p = 0.652) did not have significant impacts on the codominant comparisons. Similarly, the OM (CT vs. TT + CC: OR = 1.23, 95% CI 0.68–2.21, p = 0.495), RM (TT vs. CT + CC: OR = 0.81, 95% CI 0.30–2.20, p = 0.676), DM (CT + TT vs. CC: OR = 1.06, 95% CI 0.79–1.43, p = 0.697), and AM (T vs. C: OR = 1.00, 95% CI 0.82–1.25, p = 0.941) failed to reach statistical significance.

**Table 6:** Meta-analysis of the link between *CYP11A1* rs1484215 polymorphism with PCOS.

Model	Subgroup	Number of studies	Test of association			Test of heterogeneity			Publication bias
			OR	95% CI	P_Z_	Model	P_H_	I^2^ (%)	p-val (Egger’s test)
COD1 (CT vs. CC)	Overall	2	1.05	0.76—1.43	0.781	Fixed	0.478	0.00	NaN
COD2 (TT vs. CC)	Overall	2	1.07	0.64—1.78	0.797	Fixed	0.191	39.14	NaN
COD3 (TT vs. CT)	Overall	2	0.78	0.27—2.26	0.652	Random	0.034	77.86	NaN
DM (CT + TT vs. CC)	Overall	2	1.06	0.79—1.43	0.697	Fixed	0.790	0.00	NaN
OM (CT vs. TT + CC)	Overall	2	1.23	0.68—2.21	0.495	Random	0.083	66.68	NaN
RM (TT vs. CT + CC)	Overall	2	0.81	0.30—2.20	0.676	Random	0.039	76.65	NaN
AM (T vs. C)	Overall	2	1.00	0.82—1.25	0.941	Fixed	0.319	0.00	NaN

### False discovery rate (FDR) correction

To account for multiple testing across genetic models, the Benjamini–Hochberg false discovery rate (FDR) correction was applied to the overall meta-analysis results for SNPs that showed nominal statistical significance. For rs4077582, after FDR correction (Table S5), all previously significant associations remained statistically significant except for the codominant 3 model. For rs11632698, after FDR correction (Table S6), statistical significance was retained only in the recessive and codominant 3 models, whereas the remaining models did not remain significant following adjustment. For rs6495096, rs4887139, and rs1484215, no nominally significant associations were observed in the overall analyses; therefore, multiple testing correction was not required for these SNPs.

### Heterogeneity

The heterogeneity test for the overall population and subgroup analysis revealed low to moderate variability across studies across some genetic models, as displayed in Table 2. Significant heterogeneity for rs4077582 was identified in the COD1 (TC vs. CC) (I^2^ = 47.27%, P_H_ = 0.056), AM (T vs. C) (I^2^ = 55.77%, P_H_ = 0.015), DM (TT + TC vs. CC) (I^2^ = 42.55%, P_H_ = 0.084), and OM (TC vs. TT + CC) (I^2^ = 51.65%, P_H_ = 0.035). However, non-significant heterogeneity was detected in the cases of COD2 (TT vs. CC) (I^2^ = 17.19%, P_H_ = 0.290), COD3 (TT vs. TC) (I^2^ = 23.09%, P_H_ = 0.236), and RM (TT vs. TC + CC) (I^2^ = 14.56%, P_H_ = 0.313). Similarly, there was an indication of significant heterogeneity in the COD1 (TC vs. CC) (I^2^ = 47.98%, P_H_ = 0.073), OM (TC vs. TT + CC) (I^2^ = 46.98%, P_H_ = 0.079), and AM (T vs. C) (I^2^ = 49.95%, P_H_ = 0.062) in the Asian population. Furthermore, the Caucasian population exhibited no significant heterogeneity in any model, except for the OM (TC vs. TT + CC) model (I^2^ = 71.20%, P = 0.062). Studies using PCR–RFLP genotyping exhibited significantly high heterogeneity only in OM (TC vs. TT + CC) (I^2^ = 66.53%, P_H_ = 0.018). In contrast, ARMS-PCR genotyping revealed no heterogeneity across any genetic model (I^2^ = 0.00%), with P_H_ values of 1.000.

**Fig. 7:**
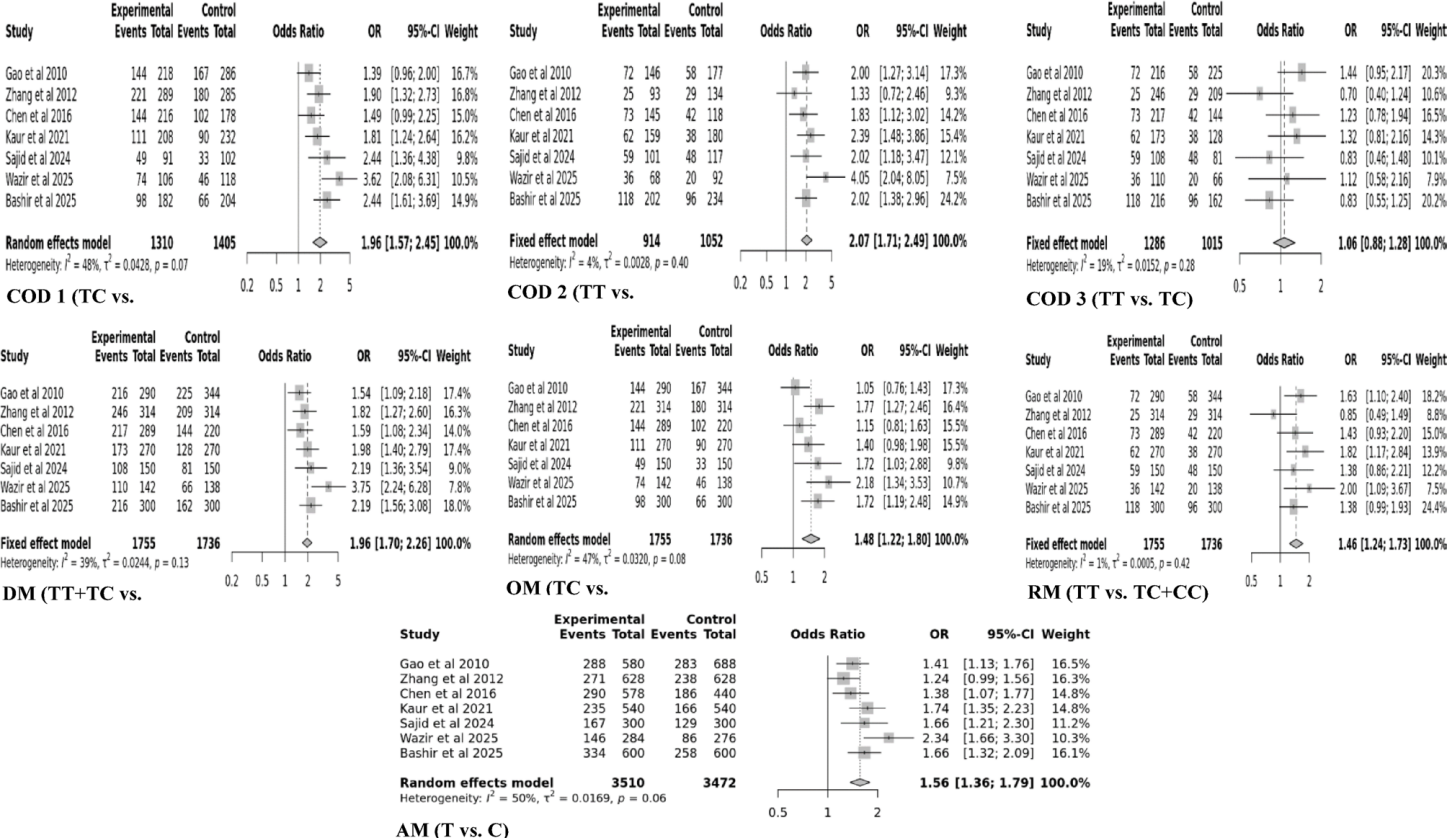
Forest plot showing the association of rs4077582 polymorphism and PCOS in perspective to Asian Populations.

**Fig. 8:**
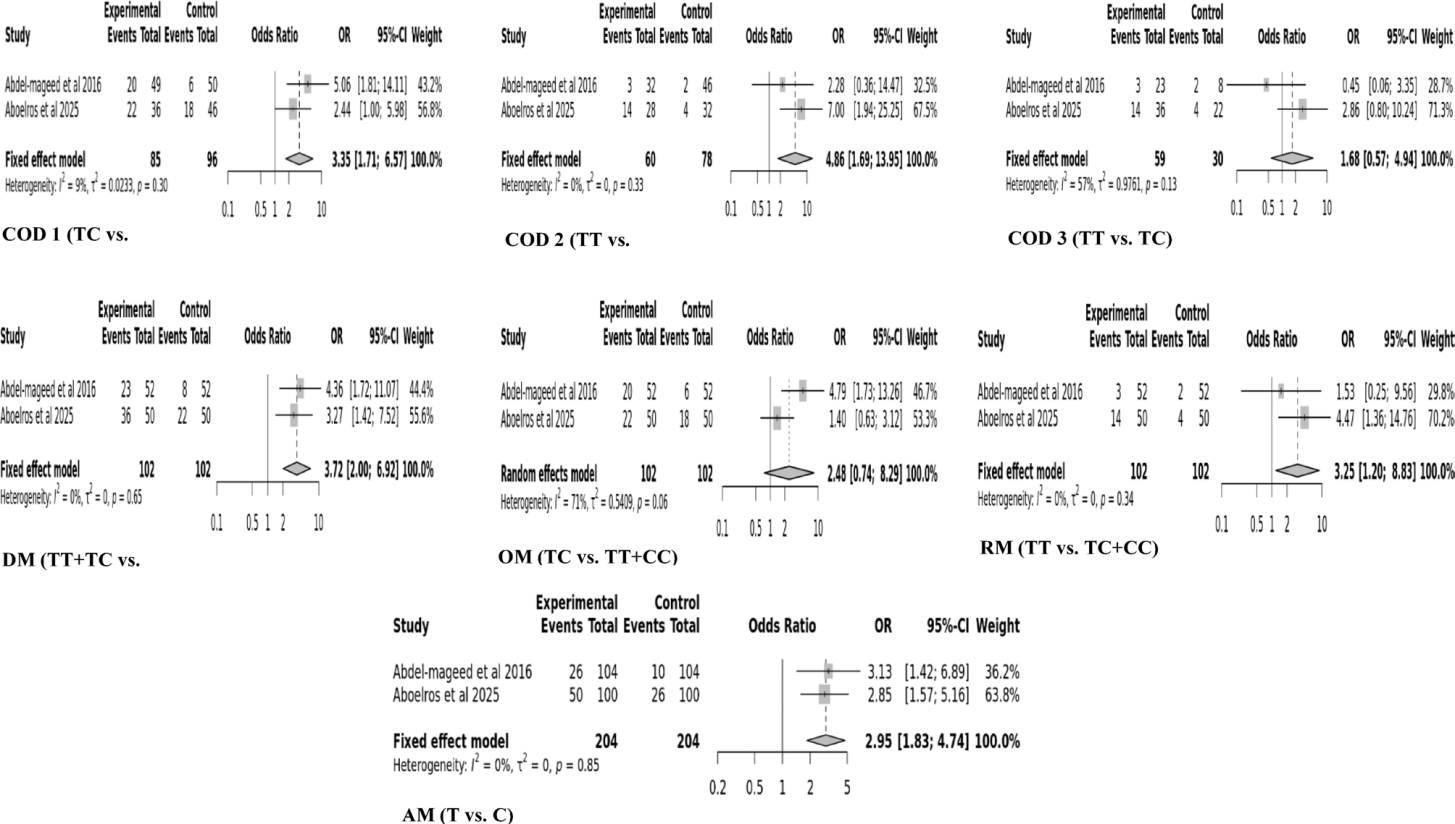
Forest plot showing the association of rs4077582 polymorphism and PCOS in perspective to Caucasian populations.

In the case of rs11632698, significant heterogeneity was noticed in all genetic models except COD3 (GG vs. GA: I^2^ = 0.00%, P_H_ = 0.628) and RM (GG vs. GA + AA: I^2^ = 28.46%, P_H_ = 0.232). The allelic model had the highest heterogeneity (G vs. A: I^2^ = 80.77%, P_H_ < 0.001), followed by the DM (GG + GA vs. AA: I^2^ = 77.38%, P_H_ = 0.001), COD1 (GA vs. AA: I^2^ = 69.64%, P_H_ = 0.011), COD2 (GG vs. AA: I^2^ = 55.85%, P_H_ = 0.060), and OM (GA vs. GG + AA: I^2^ = 71.39, P_H_ = 0.007). On the other hand, significant heterogeneity was observed in numerous genetic models for rs6495096. The OM (CG vs. GG + CC: I^2^ = 82.44%, P_H_ = 0.003), COD1 (CG vs. CC: I^2^ = 77.68%, P_H_ = 0.011), and DM (GG + CG vs. CC: I^2^ = 72.72%, P_H_ = 0.026) all showed significant heterogeneity. On the other hand, heterogeneity was not detected observed in codominant Model 3 (GG vs. CG: I^2^ = 42.54%, P_H_ = 0.176), the RM (GG vs. CG + CC: I^2^ = 27.81%, P_H_ = 0.250), COD2 (GG vs. CC: I^2^ = 0.00%, P_H_ = 0.454), or AM (G vs. C: I^2^ = 10.56%, P_H_ = 0.327) (Figs. 7 and 8).

**Fig. 9:**
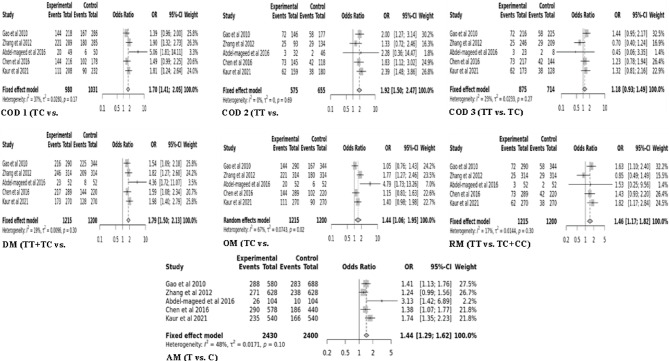
Forest plot showing the association of rs4077582 polymorphism and PCOS in perspective to PCR–RFLP method.

**Fig. 10:**
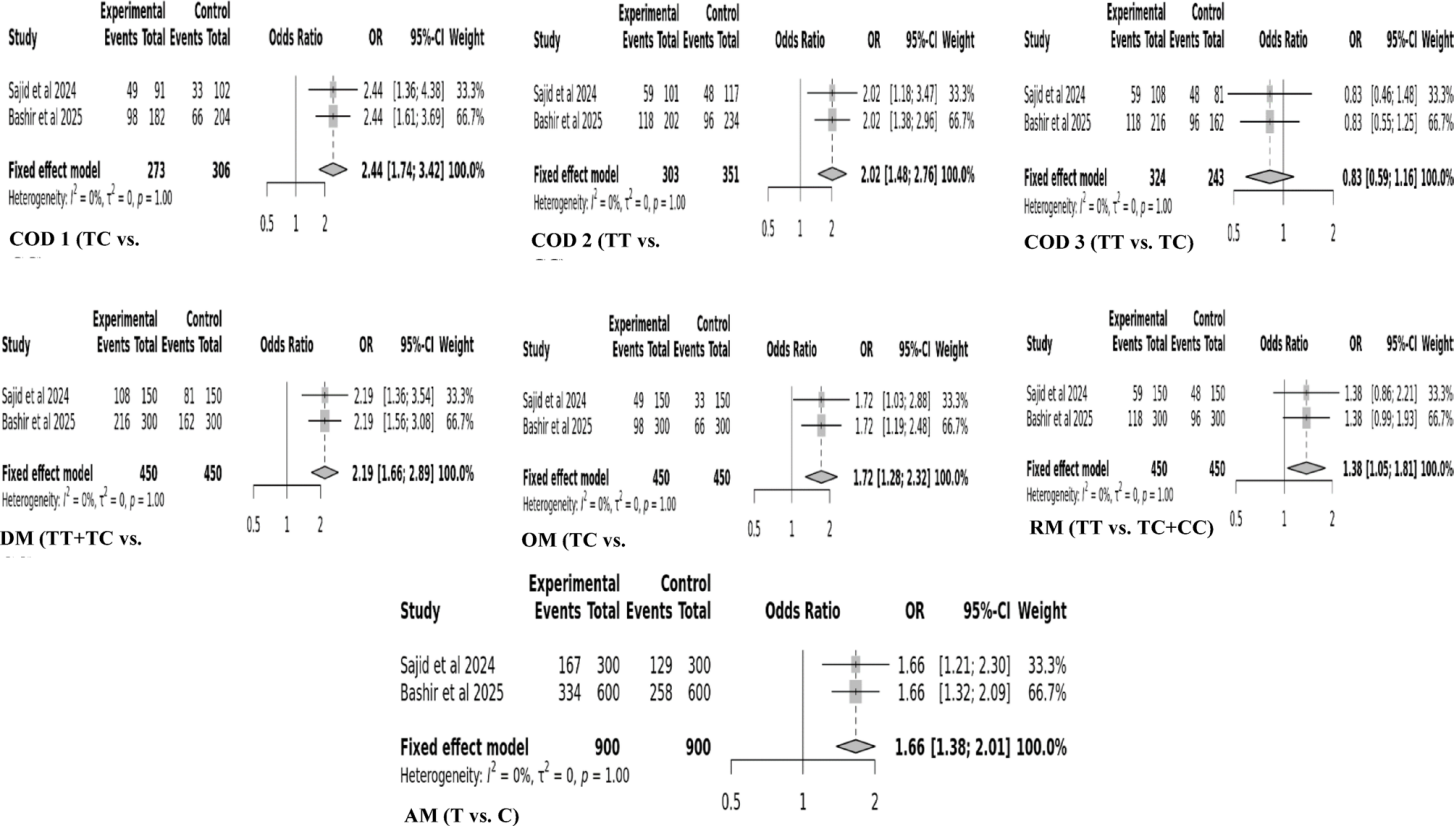
Forest plot showing the association of rs4077582 polymorphism and PCOS in perspective to ARMS-PCR method.

Except for COD1** (**AG vs. AA: I^2^ = 0.00%, P = 0.828), most models exhibited significant moderate to high heterogeneity in the case of rs4887139. Significant heterogeneity was demonstrated by the COD2 (GG vs. AA: I^2^ = 71.45%, P_H_ = 0.030), 3 (GG vs. GA: I^2^ = 84.28%, P_H_ = 0.002), OM (AG vs. GG + AA: I^2^ = 85.27%, P_H_ = 0.001), RM (GG vs. AG + AA: I^2^ = 86.84%, P_H_ < 0.001), and AM (G vs. A: I^2^ = 88.24%, P_H_ < 0.001). In certain genetic models, significant heterogeneity was found for rs1484215. Significant heterogeneity was demonstrated by COD3 (TT vs. CT: I^2^ = 77.86%, P_H_ = 0.034), OM (CT vs. TT + CC: I^2^ = 66.68%, P_H_ = 0.083), and RM (TT vs. CT + CC: I^2^ = 76.65%, P_H_ = 0.039). The COD1 (CT vs. CC: I^2^ = 0.00%, P_H_ = 0.478), COD2 (TT vs. CC: I^2^ = 39.14%, P_H_ = 0.191), DM (CT + TT vs. CC: I^2^ = 0.00%, P_H_ = 0.790) and AM (T vs. C: I^2^ = 0.00%, P_H_ = 0.319) did not exhibit any heterogeneity (Figs. 9 and 10).

### Publication bias and sensitivity analysis

**Fig. 11:**
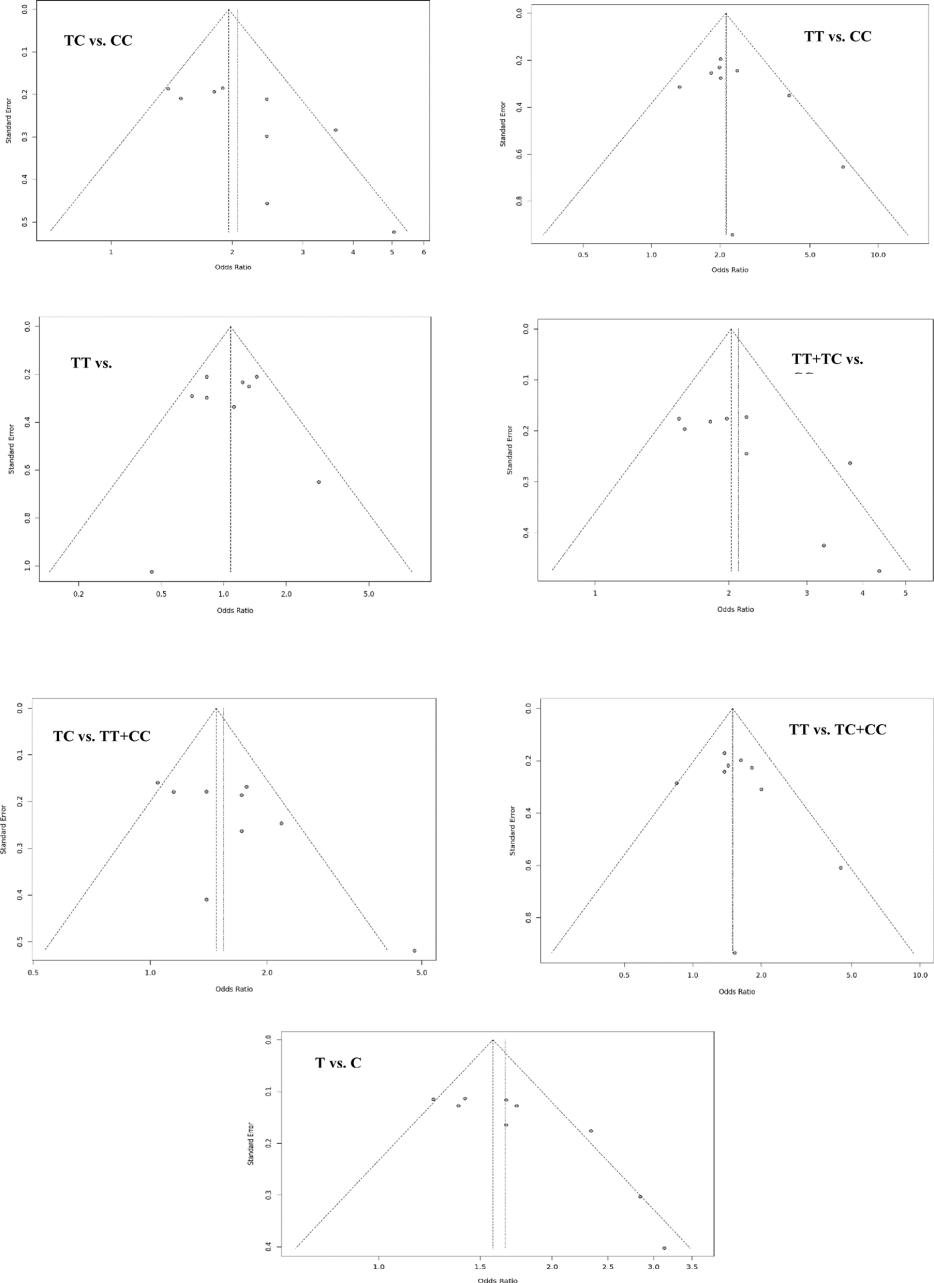
Funnel plot showing publication bias for detection of association of rs4077582 with PCOS.

**Fig. 12:**
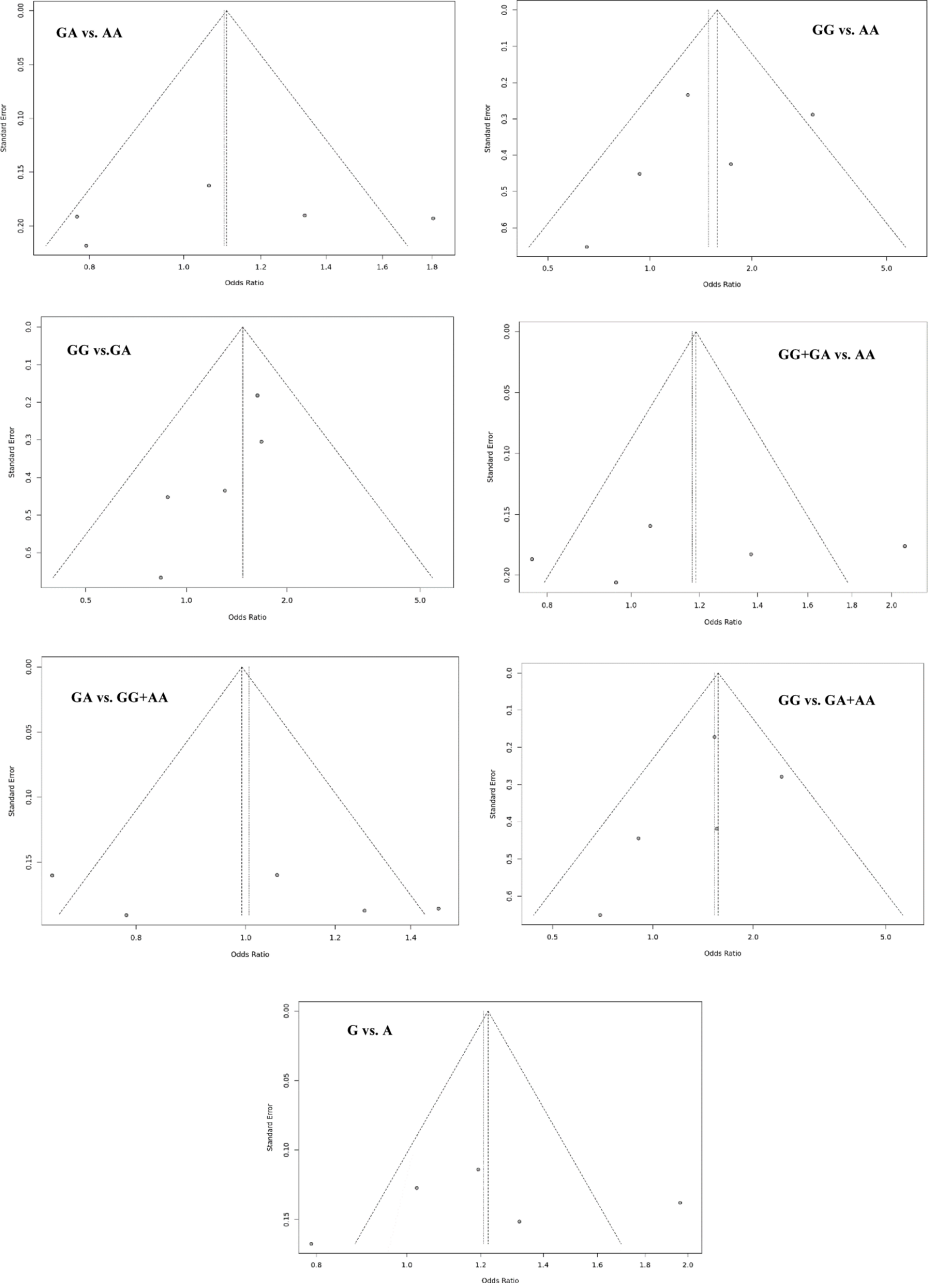
Funnel plot showing publication bias for detection of association of rs11632698 with PCOS.

**Fig. 13:**
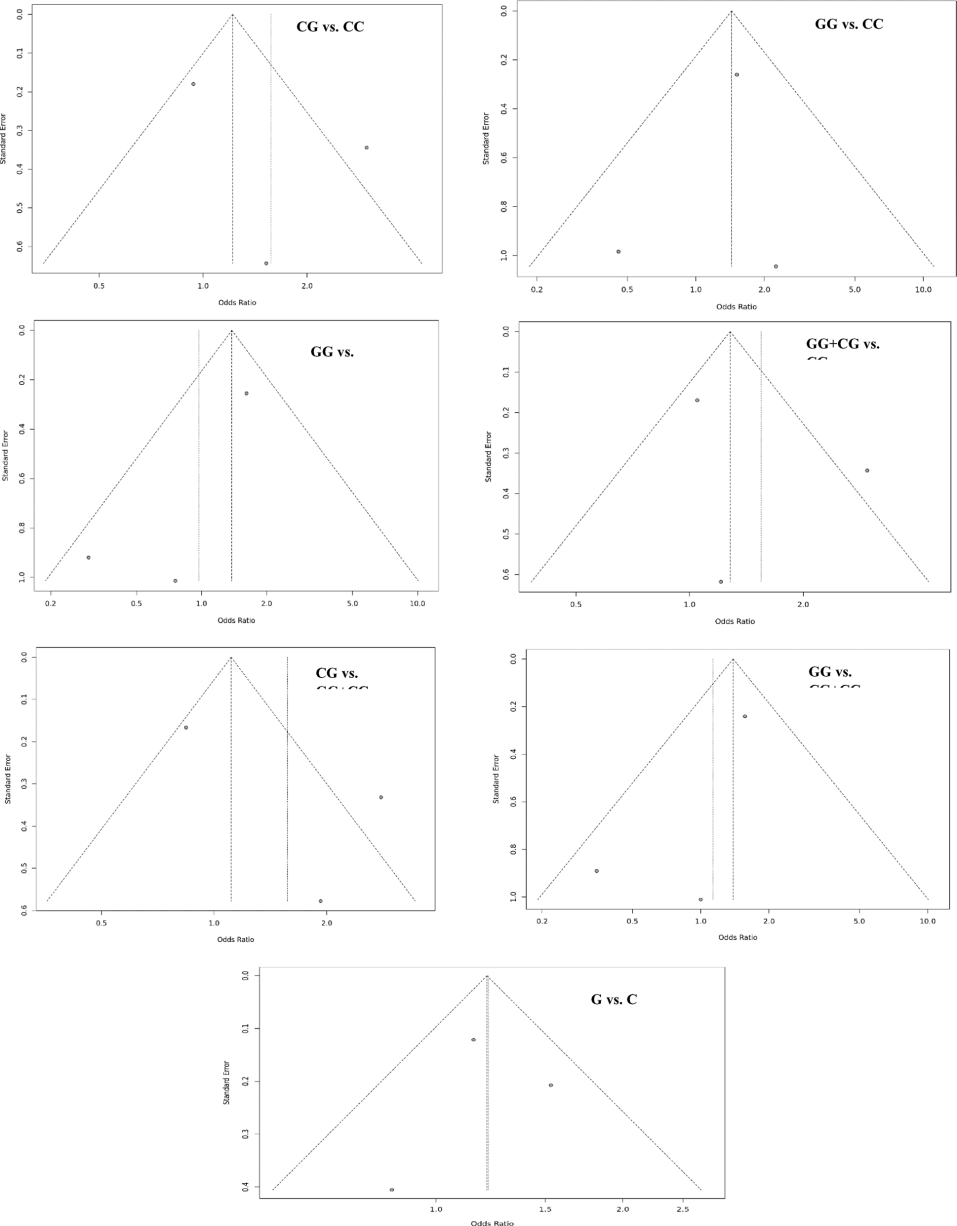
Funnel plot showing publication bias for the detection of the association of rs6495096 with PCOS.

**Fig. 14:**
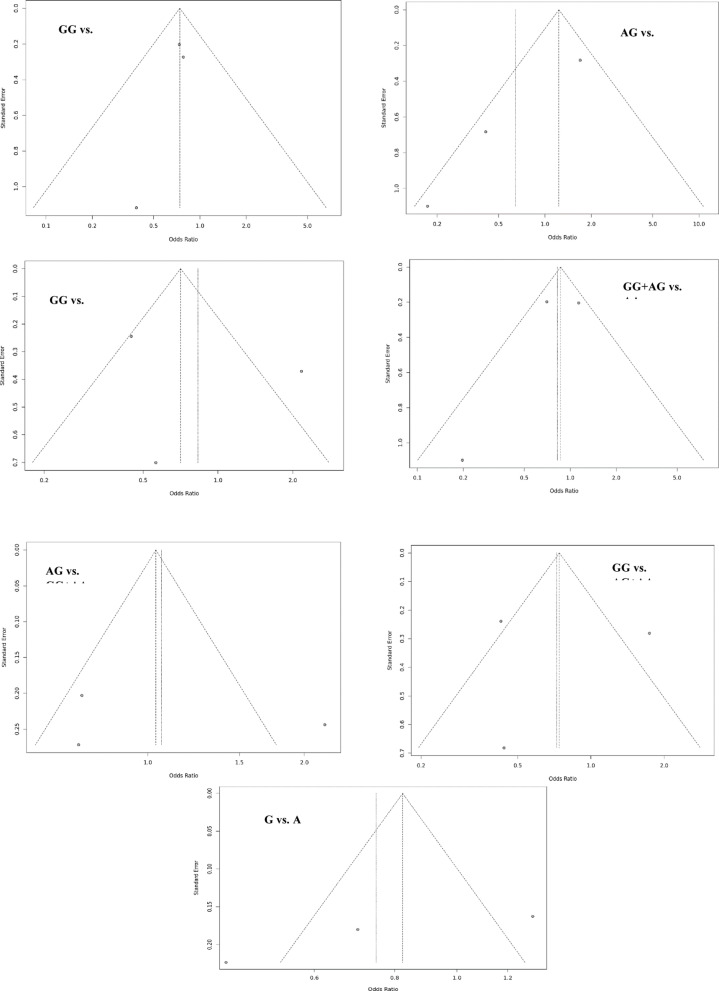
Funnel plot showing publication bias for the detection of the association of rs4887139 with PCOS.

**Fig. 15:**
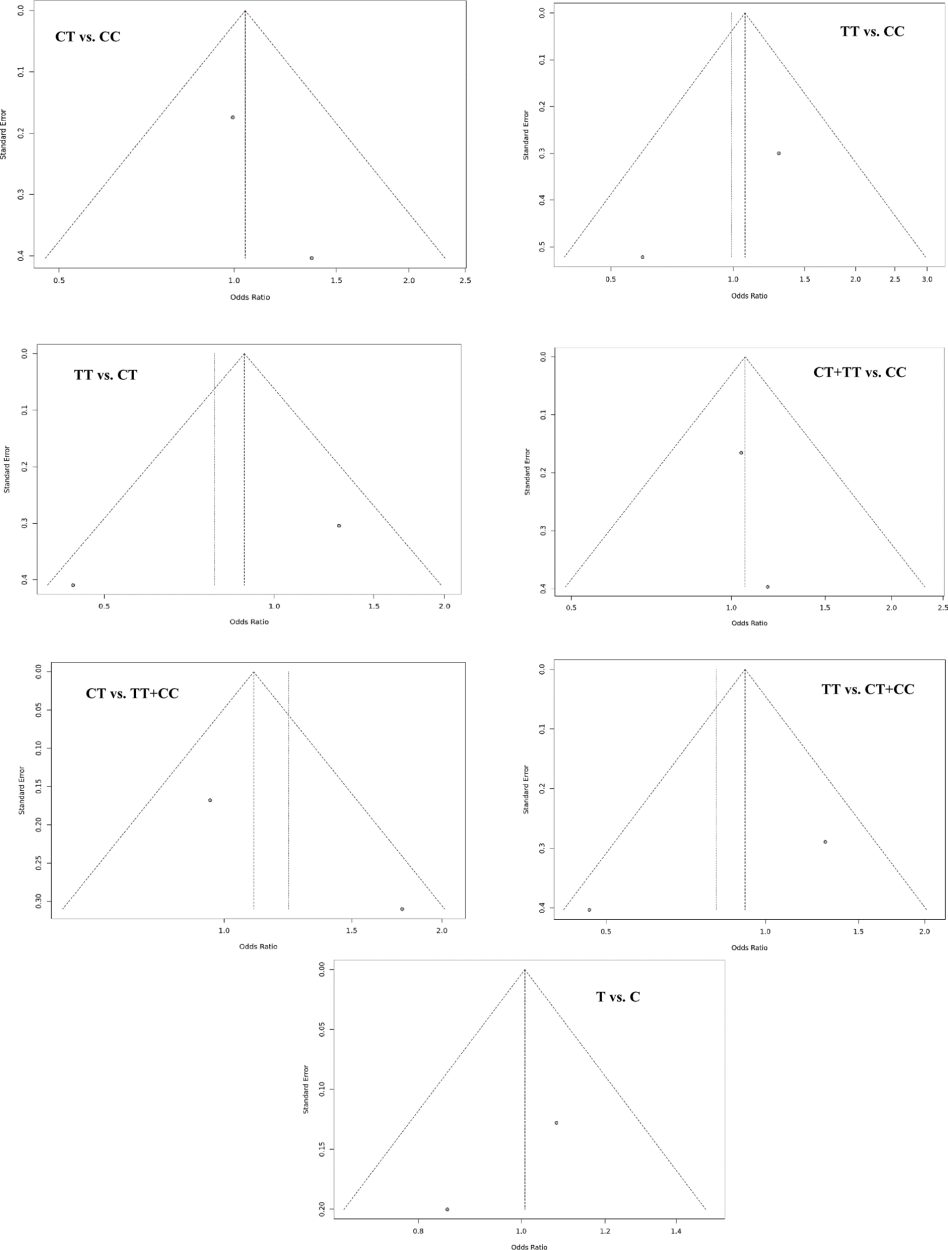
Funnel plot showing publication bias for the detection of the association of rs1484215 with PCOS.

**Fig. 16:**
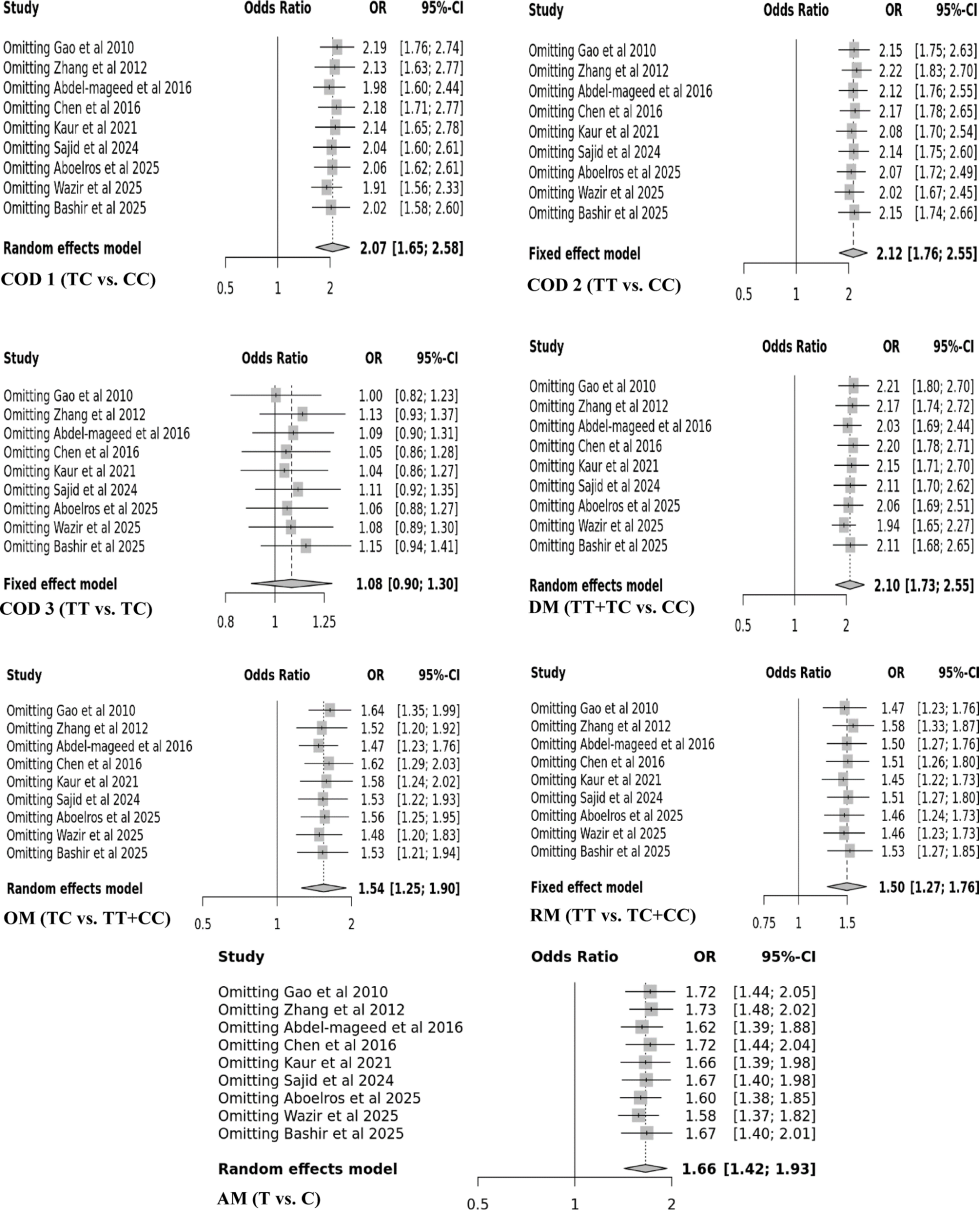
Sensitivity analysis plots for the association of rs4077582 and PCOS in different genetic models.

**Fig. 17:**
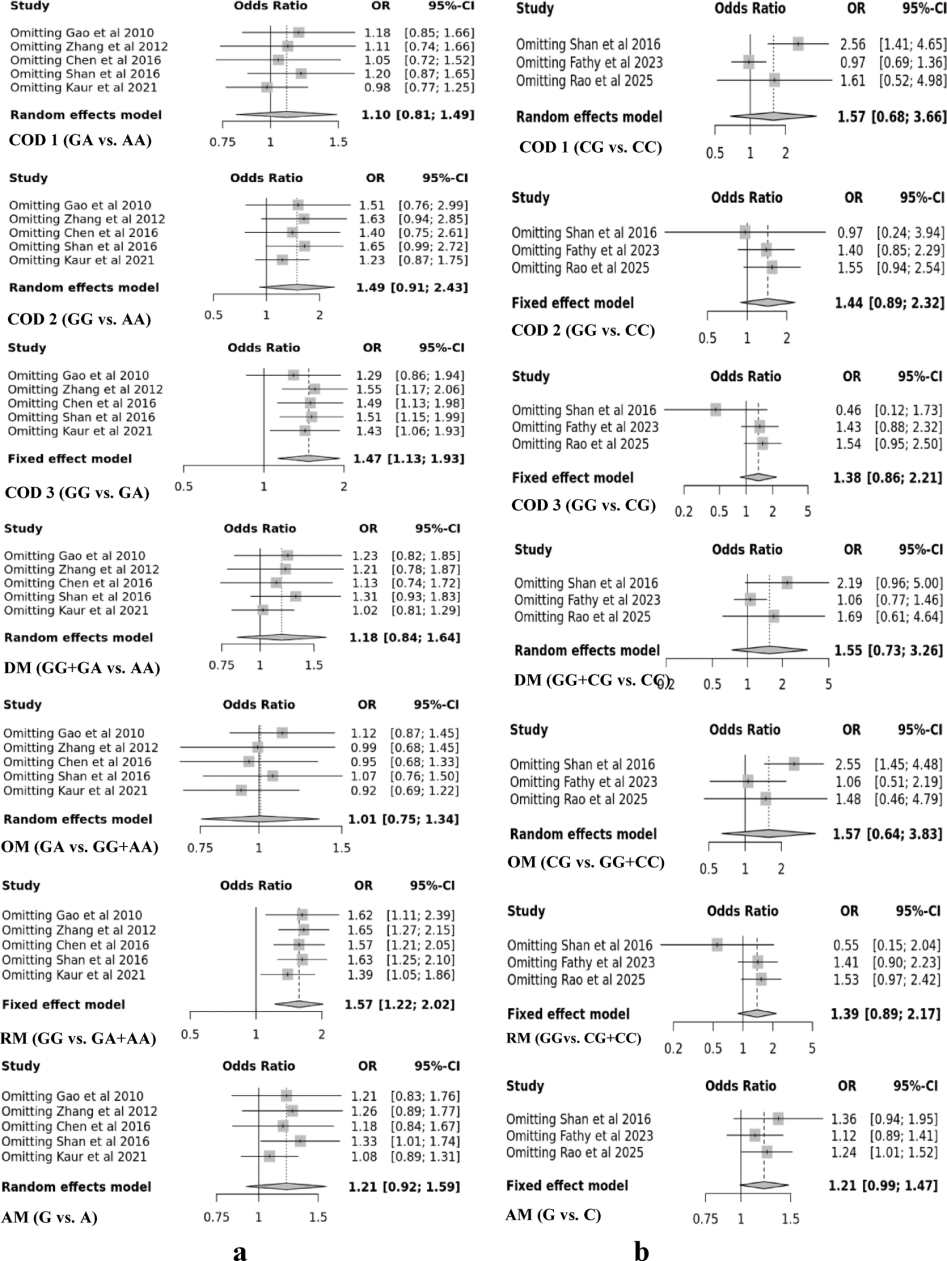
Sensitivity analysis plots for the association of **(a)** rs11632698 and **(b)** rs6495096 with PCOS in different genetic models.

**Fig. 18:**
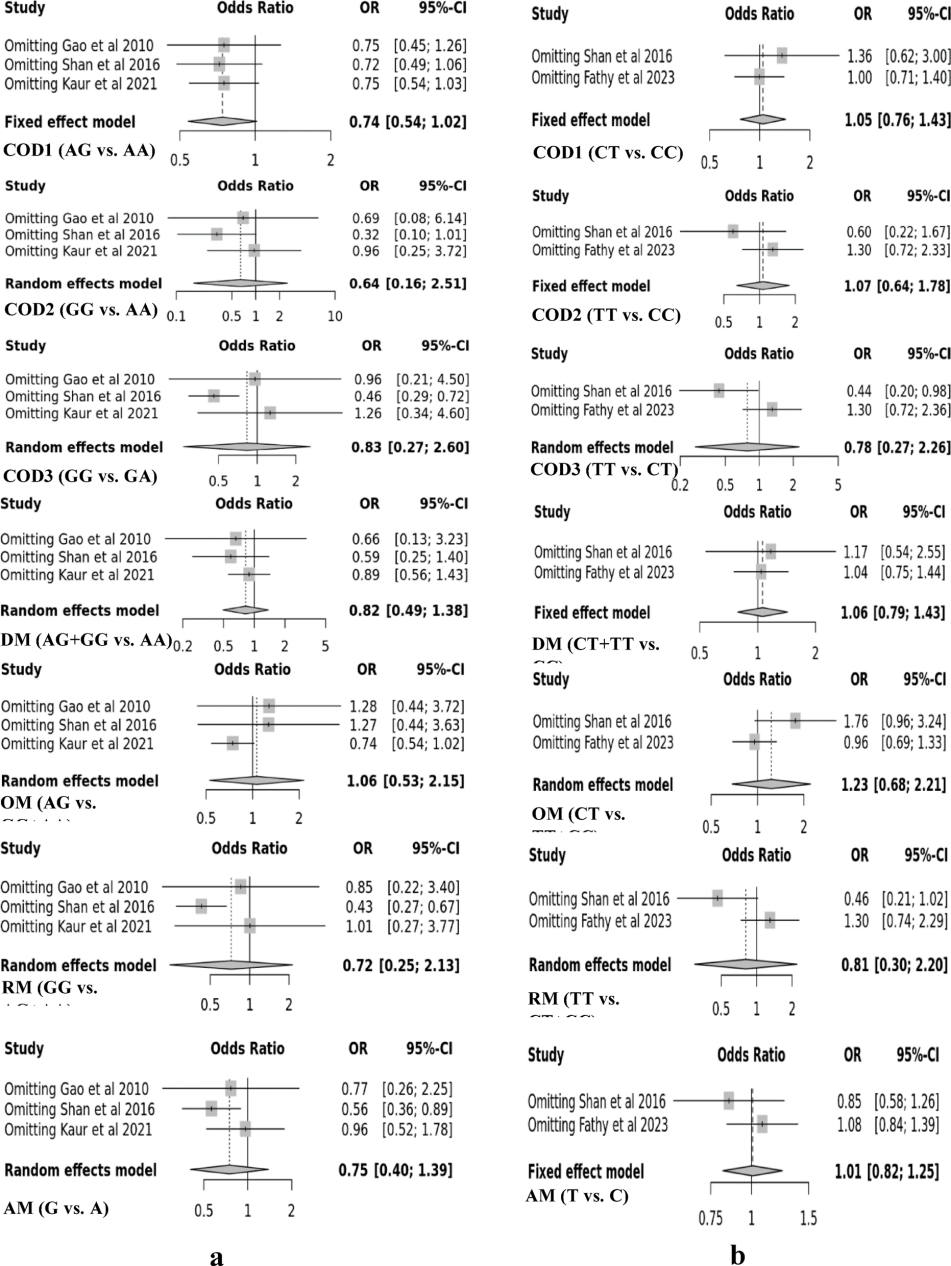
Sensitivity analysis plots for the association of **(a)** rs4887139 and **(b)** rs1484215 with PCOS in different genetic models.

Figure 11 displays the funnel plots, whereas Table 2 presents the bias parameters of Egger’s test for rs4077582. The bias assessment was performed across all studies, revealing that there was a lack of the funnel plot’s apparent asymmetry for COD2, COD3, OM, or RM, indicating that publication bias did not exist. The remaining three models suggested the presence of publication bias (p value < 0.05). Because Egger’s test indicated potential small-study effects for rs4077582 in the COD1 (p = 0.039), DM (p = 0.026), and AM (p = 0.013) models, a trim-and-fill analysis was performed using the Duval and Tweedie nonparametric method under a random-effects model. The procedure imputed three potentially missing studies for COD1 and two studies for both DM and AM models. After adjustment, the pooled odds ratios were slightly attenuated but remained statistically significant: COD1 (OR = 1.83, 95% CI 1.43–2.33), DM (OR = 1.92, 95% CI 1.54–2.41), and AM (OR = 1.58, 95% CI 1.34–1.85), all p < 0.001. These findings indicate that the association between rs4077582 and PCOS risk remains robust even after correction for potential publication bias (Table S4). For rs11632698, Egger’s test revealed no indication of publication bias across any genetic model (Table 3), and funnel plots (Fig. 12) revealed no significant asymmetry. Similarly, the funnel plot and Egger test demonstrated no evidence of publication bias in any model for rs6495096 (Table 4 and Fig. 13) or rs4887139 (Table 5 and Fig. 14). Egger’s test was not applicable for rs1484215 because of insufficient number of studies for a meaningful test, but the funnel plot revealed the absence of publication bias (Fig. 15). To ensure credibility, we excluded every case–control study to perform a sensitivity analysis sequentially for every SNP. No major distinctions were detected in the pooled ORs of any of the genetic models for any of the included SNPs, indicating the robustness and stability of all the findings (Figs. 16, 17, and 18). Additionally, for rs4077582, sensitivity analysis excluding HWE-violating studies showed that all genetic models remained statistically significant, except for OM (Table S2). Although associations remained significant after excluding HWE-violating studies, borderline publication bias persisted in some models, suggesting that these findings should be interpreted with caution.

### TSA results

**Fig. 19:**
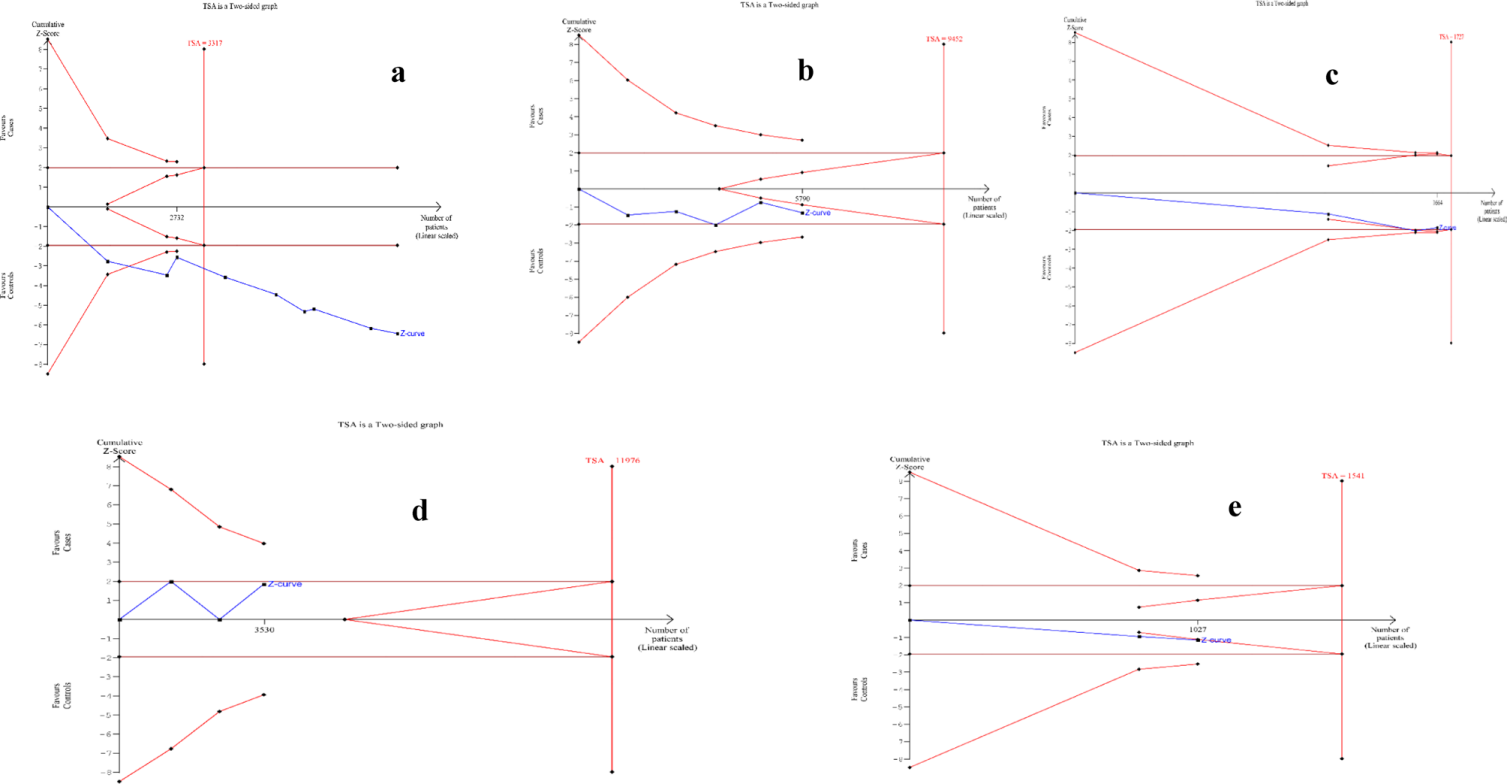
Trial sequential analysis for **(a)** rs4077582 in AM (T vs. C), **(b)** rs11632698 in AM (G vs. A), **(c)** rs6495096 in AM (G vs. C), **(d)** rs4887139 (G vs. A), and **(e)** rs1484215 (T vs C).

Under the predefined assumptions (5% type I error, 80% power, and 20% anticipated relative risk reduction), the cumulative Z-curve for rs4077582 in the allelic model crossed both the conventional and trial sequential monitoring boundaries, and the accrued sample size exceeded the required information size (RIS), suggesting statistical stability of the association within the specified assumptions (Fig. 19a). However, because TSA was originally developed for randomized controlled trials, interpretation in observational genetic meta-analyses should be approached cautiously and does not eliminate the need for further independent validation.

In contrast, for rs11632698, rs6495096, rs4887139, and rs1484215 (Figs. 19b–e), the cumulative Z-curves did not cross the monitoring boundaries nor reach the RIS, indicating that the currently available evidence remains insufficient under the predefined assumptions and that additional well-designed studies are required.

### In silico CYP11A1 gene expression

**Fig. 20:**
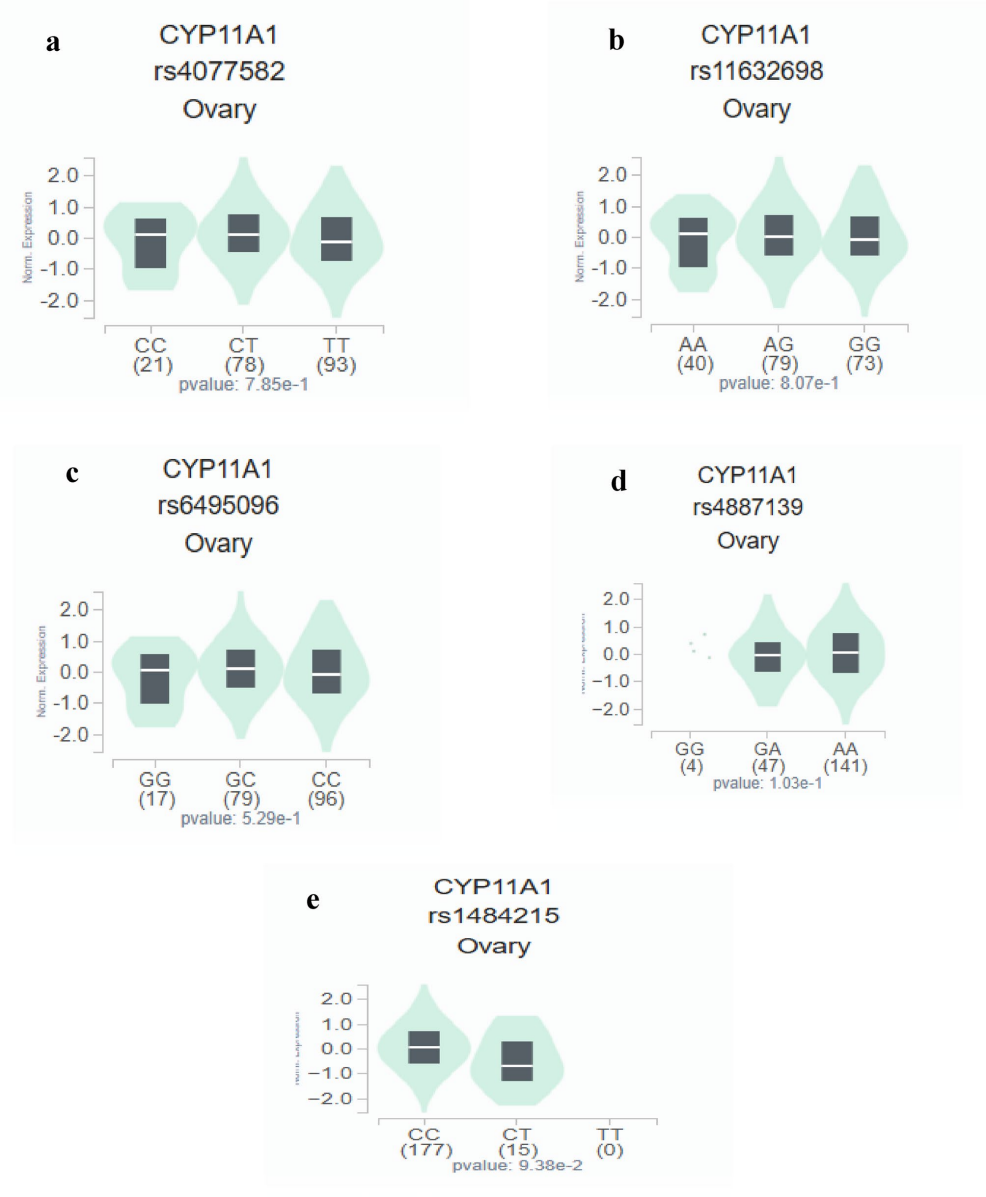
In silico expression analysis of CYP11A1 in relation to different genotypes of the rs4077582 **(a)**, rs11632698 **(b)**, rs6495096 **(c),** rs4887139 **(d)**, and rs1484215 **(e)** polymorphisms. The values in the brackets represent the frequency of certain genotype carriers.

Based on the data from the GTEx portal, increased expression of CYP11A1 mRNA in ovary tissues was connected to the heterozygous genotypes of rs4077582 (p = 0.785), rs11632698 (p = 0.807), and rs6495096 (p = 0.529), whereas the heterozygous genotypes of rs4887139 (p = 0.103) and rs1484215 (p = 0.0938) were responsible for the reduced expression of CYP11A1 **(**Fig. 20**)**. Moreover, the generated violin plots demonstrated that none of the five CYP11A1 SNPs presented significant variations in mRNA expression between the three normal homozygous (NH), heterozygous (HE), and mutant homozygous (MH) genotypes.

## Discussion

Genetic advancements have demonstrated substantial reprogramming of all cellular pathways involved in the progression of PCOS^42–44^. It is believed that PCOS is prompted by genes that produce the steroidogenic pathway’s enzymes. Among them, the CYP11A, CYP17, and CYP19 genes are the most recognized^45^. CYP11A1 is implicated in the pathogenesis of PCOS, with its polymorphisms linked to modified steroidogenic activity that may lead to elevated androgen levels characteristic of PCOS^20,23^. PCOS is prominently characterized by disordered steroidogenesis, which may result from CYP11A1 mutations that affect cholesterol conversion^46^. Thus, increased CYP11A1 gene expression may result in improved conversion of cholesterol to pregnenolone, leading to overproduction of androgens —a hallmark of PCOS^45^. Similarly, a study revealed that CYP11A1 polymorphisms may have a modest impact on PCOS development and serve as genetic risk markers for hyperandrogenic phenotypes^47^. Moreover, numerous earlier investigations on CYP11A1 have shown a notable association between PCOS risk and frequently encountered polymorphisms in the CYP11A1 locus^25,48^. In contrast, no substantial associations were found in some studies^15,20,34^. This is, to the best of our understanding, the first meta-analysis conducted to determine the association between PCOS risk and common *CYP11A1* gene variants (rs4077582, rs11632698, rs6495096, rs4887139, and rs1484215). It simultaneously evaluates five SNPs within a single analytical framework, applies trial sequential analysis (TSA) to assess conclusiveness, and integrates functional in silico analyses. Among Asian and Caucasian populations, twelve case–control studies totaling 2267 PCOS-affected individuals and 2262 healthy controls were added to this meta-analysis.

Among the five examined CYP11A1 genetic variations, rs4077582 is the most studied polymorphism for PCOS association studies and is significantly associated with PCOS risk in both Asian^15,22,27,29,30,32,33^ and Caucasian^28,31^ populations. In support of these studies, our study revealed that the T allele was associated with a 1.69-fold greater risk of developing PCOS compared to the C allele. Similarly, this polymorphism is significantly associated with PCOS in Pakistani^32^, Indian^22^, and Egyptian women^31^, with ORs indicating a 1.30-, 1.72-, and 3.72-fold greater vulnerability to PCOS in T allele carriers than in C allele carriers, respectively. Our study also revealed that the heterozygous genotype (CT) and mutant genotype (TT) significantly increased PCOS susceptibility by 2.16- and 2.12-fold, respectively. Research in North Indian populations also supports our findings, revealing that the (CT) and (TT) genotypes significantly increase the incidence of PCOS by 1.79 and 1.96 times, respectively. Similarly, the Egyptian cohort also revealed that the heterozygous (CT) and mutant (TT) can significantly increase the susceptibility to PCOS by 3.45- and 6.3-fold, respectively, establishing this SNP as a possible genetic marker for PCOS propensity in overall populations. Only significant heterogeneity was identified in the allele model for overall populations in our meta-analysis, indicating potential study-level differences in the association under only the allelic model. Moreover, sensitivity analysis excluding six studies with HWE violations indicates that the overall association between CYP11A1 rs4077582 and PCOS risk remained largely robust, but genetic model-specific associations may be partially influenced by genotyping error, population structure, control selection, and study quality.

Although the strength of the association varied, our subgroup analysis manifested that the examined polymorphism was connected to PCOS susceptibility in both the Asian and Caucasian groups. Multiple models (codominant, dominant, recessive, and allelic) revealed significant relationships in Asian cohorts, with odds ratios suggesting a slight increase in PCOS risk. However, although only two studies were available, the pooled analysis indicated larger impact sizes in Caucasian cohorts, especially in the TT vs. CC and allelic models, where the odds ratios were significantly greater than those for Asians. However, a review on steroidogenesis highlighted that polymorphisms in steroidogenic genes (e.g., CYP11A1, CYP17, and CYP19) confer a moderate risk for PCOS in both Asian and Caucasian populations, reflecting a potential population-specific effect driven by allele frequency differences and the hormonal milieu^23^. Moreover, the increased and significant risk association in both Asian and Caucasian studies may reflect a higher prevalence of the T allele, which is linked to increased CYP11A1 expression and androgen production, a hallmark of PCOS^49,50^. Most importantly, rs4077582 (C > T) has been found to be significantly associated with the occurrence of PCOS in various populations, including Indian^22^, Chinese^15,27,29^, Egyptian^28,31^, and Pakistani^30,32,33^ individuals. These studies suggest that rs4077582 is consistently related to increased vulnerability to PCOS; however, the extent of this association may vary by ethnic background. Heterogeneity was observed in COD1, OM, and AM for Asian studies, indicating inconsistent findings across these models. In contrast, although heterogeneity seemed to be present only in OM for the Caucasian subgroup, the small number of studies precluded definitive conclusions. The subgroup analysis among Caucasians suggested a possible trend toward association; however, the results are based on only two studies with a limited number of participants, leading to low statistical power and wide confidence intervals. Therefore, these findings should be regarded as exploratory and interpreted cautiously until further data from larger, independent cohorts become available. However, despite both subgroups showing a significant association, the evidence is stronger and more reliable for Asian populations because more studies are available.

The subgroup analysis of rs4077582, conducted via the genotyping approach, revealed significant differences in the intensity and consistency of association between the investigated polymorphism and PCOS susceptibility. The PCR–RFLP subgroup showed significant relationships in most models, with the codominant (OR = 1.93) and dominant (OR = 1.79) models exhibiting the largest effect sizes. Consistent with our meta-analysis findings, all research using the PCR–RFLP method has revealed a significant association with PCOS in India^22^, China^15,27,29^, and Egypt^28^. No significant relationships were found in our study for the codominant 3 model in the case of either PCR–RFLP or ARMS-PCR, suggesting model-specific variability in detecting genetic risk. Notably, ARMS-PCR studies demonstrated no heterogeneity, whereas studies using PCR–RFLP exhibited significant heterogeneity in only one of the OM, indicating that the observed associations are consistent across genotyping platforms and support a dominant or additive model of inheritance for the risk allele (T) in the development of PCOS. However, methodological issues, such as genotyping accuracy and demographic characteristics, may contribute to the observed heterogeneity, necessitating further exploration in larger and multicenter studies.

Only five studies have explored the role of the rs11632698 SNP in PCOS susceptibility in Asian women, among which one study reported a statistically significant interaction, with higher susceptibility outcomes in the Indian population^22^. In contrast, four other studies in Chinese populations did not find a significant association^15,20,27,29^. A North Indian study revealed that individuals with the rs11632698 risk G allele had a nearly twofold greater risk (OR = 1.98) of developing PCOS^22^, whereas our meta-analysis indicated that this G allele increases the risk of PCOS by 1.17 times, but this evidence is not statistically significant. Our meta-analysis result, however, presented that this SNP is not substantially linked to PCOS in Asian people. Since all the studies on rs11632698 were conducted in Asian populations, no interethnic comparisons were possible in our study. The high heterogeneity and lack of a meaningful association suggest that, unlike rs4077582, rs11632698 may not be a major contributor to the vulnerability of Asian populations to PCOS. However, the high heterogeneity across studies indicates significant variety in effect sizes. Significant heterogeneity between studies can lead to non-replicability thresholds, making it difficult to reach sufficient statistical power, regardless of sample size^53^.

A noteworthy finding in the literature is that the effect of rs6495096 on PCOS risk varies by ethnic group. rs6495096 has been shown to possess potent and noteworthy defensive effects against PCOS in Iranian women^21^, but a lack of significant effects on PCOS has been reported in Chinese and Pakistani women^20,34^. Our meta-analysis revealed that the GG genotype may increase PCOS susceptibility by 1.44-fold, although this increase was not statistically significant. Consistent with our findings, GG genotypes were linked with a 1.51-fold elevated likelihood of PCOS in Chinese women^20^. In conclusion, rs6495096 did not show a statistically significant association with PCOS probability in the Asian population for any of the genetic models tested in our meta-analysis. The rs6495096 also showed significant heterogeneity in several models, such as the overdominant and codominant model 1. However, certain models, including the allelic and recessive models, displayed no heterogeneity, suggesting more stable findings compared to rs11632698.

To investigate the association between the rs4887139 polymorphism in the CYP11A1 gene and susceptibility to polycystic ovary syndrome (PCOS), this meta-analysis combined data from only three studies. None of the pooled associations were statistically significant, despite the majority of genetic models indicating a possible elevated risk. In line with our findings, a study in China also demonstrated that this SNP is not substantially linked to PCOS^20^. However, Kaur et al. revealed that this polymorphism is associated with an increased risk of PCOS in Indian women^22^, whereas another study by Gao et al. revealed the protective effect of this SNP against the development of PCOS in Chinese women^27^. However, the high heterogeneity in most models in our findings suggests variability in effect sizes. According to a pair of studies, this meta-analysis examined the relationship between the rs1484215 polymorphism and the risk of PCOS. Neither of the genetic models demonstrated a statistically significant association overall in our meta-analysis, as these included two studies that failed to reach significant outcomes in Chinese^20^ and Iranian women^21^.

Considerable heterogeneity was observed across several SNPs and genetic models, particularly for rs11632698 and rs4887139, where I^2^ values exceeded 70%. This variability likely reflects differences among studies in ethnic composition, study design, control selection (hospital- vs population-based), diagnostic criteria for PCOS (e.g., NIH, Rotterdam, or AE-PCOS), participant characteristics such as BMI and age, matching variables, control definitions, and genotyping techniques used to detect CYP11A1 variants^51,52^. Although subgroup analysis by ethnicity for rs4077582 were conducted, data limitations prevented the performance of formal meta-regression or stratified analyses by diagnostic criteria, BMI, or genotyping method. Therefore, results from models showing high heterogeneity should be interpreted with caution, as they may reflect methodological or population-level variability rather than true genetic differences. Publication bias was minimal for rs4077582, with only a few models suggesting potential bias, whereas no evidence of bias was found for rs11632698, rs6495096, and rs4887139 across any model, as confirmed by Egger’s test and symmetrical funnel plots. After applying trim-and-fill correction in models with significant Egger’s test results, the adjusted pooled odds ratios remained statistically significant despite mild attenuation of effect size. This indicates that potential small-study effects did not materially alter the overall association between rs4077582 and PCOS susceptibility. Egger’s test was not applicable for rs1484215 because there were insufficient studies included in our analysis. However, Egger’s regression test has low statistical power when fewer than 10 studies are included, and publication bias assessments should therefore be interpreted cautiously^39^. Sensitivity analysis further supported the reliability of the results across all SNPs; the sequential exclusion of studies did not cause any major fluctuations in the overall outcomes, demonstrating the consistency and continuity of the results. Trial sequential analysis (TSA) suggests that, under the predefined assumptions, the association between the CYP11A1 rs4077582 polymorphism and PCOS appears statistically stable; however, as TSA was originally developed for randomized trials, its interpretation in observational genetic meta-analyses should remain cautious and does not eliminate the need for further independent validation. Moreover, rs11632698, rs6495096, rs4887139, and rs1484215 did not reach either threshold, so these findings have been interpreted as inconclusive rather than definitive associations, indicating insufficient evidence and the need for further studies. The in-silico eQTL analysis did not reveal statistically significant changes in CYP11A1 mRNA expression associated with the analyzed SNPs in ovarian tissue. This suggests that the genetic variants examined may not exert strong cis-regulatory effects detectable within the GTEx dataset, or that current sample sizes are insufficient to capture subtle expression differences. Therefore, these findings should be interpreted as exploratory and hypothesis-generating, rather than definitive evidence of altered gene expression. Earlier studies have proposed potential functional impacts of CYP11A1 polymorphisms on transcriptional regulation^22,24,29^; however, our current GTEx-based in-silico results do not corroborate such effects in ovarian tissue. Therefore, the functional effect of these variants on CYP11A1 expression remains uncertain and requires further experimental validation.

Considering the results of this study, several limitations should be acknowledged. First, a relatively small number of case–control studies were included in the meta-analysis, and the overall sample size of patients and controls was limited. This may reduce statistical power and affect the precision of pooled estimates. Second, nine of the twelve included studies were conducted in Asian populations, with only three from Caucasian populations, potentially introducing ethnic imbalance and limiting generalizability to other populations such as African or Hispanic groups. Third, five SNPs were evaluated under seven genetic models (35 total comparisons), which may increase the risk of Type I error due to multiple testing. To mitigate this concern, the allelic (additive) model was defined a priori as the primary genetic model, and Benjamini–Hochberg false discovery rate (FDR) correction was applied to SNPs showing nominal statistical significance. After correction, associations for rs4077582 remained robust, whereas rs11632698 demonstrated significance only in specific secondary models. Therefore, results from non-primary models should be interpreted as exploratory and warrant further validation. Additionally, most included studies used hospital-based controls rather than population-based controls, which may introduce selection bias and potentially inflate effect estimates. Furthermore, although in silico genotype-based expression analyses were performed, no experimental in vitro functional validation was conducted to confirm genotype-dependent differences in CYP11A1 expression between PCOS patients and controls.

Moreover, TSA was conducted using predefined assumptions, including a 20% anticipated relative risk reduction, which may not fully reflect the smaller effect sizes typically observed in observational genetic studies; therefore, TSA findings should be interpreted cautiously.

Despite these limitations, the present meta-analysis, supported by trial sequential analysis (TSA), provides supportive evidence of a potential genetic contribution of CYP11A1 variants to PCOS susceptibility, particularly rs4077582. Based on the Venice criteria assessment, the cumulative evidence supporting rs4077582 as a susceptibility locus for PCOS is moderate to strong; however, replication in larger, multi-ethnic cohorts and functional validation studies remains necessary. Large-scale, multi-ethnic studies and functional investigations are required to confirm causality and clarify gene–environment interactions before translation into precision medicine strategies.

## Conclusion

Among the polymorphisms examined, CYP11A1 rs4077582 was detected as an inherent increased risk factor for PCOS, demonstrating consistent and statistically significant associations across genotyping techniques and models. In addition, our findings suggest a possible association of rs11632698 with PCOS susceptibility under codominant 3 and recessive models; however, TSA results indicate that additional studies are needed to reach firm conclusions. In contrast, the rs4887139 and rs1484215 were found to have no significant impact on PCOS susceptibility. Moreover, our results highlight the crucial role of rs4077582 in the pathogenic mechanism of PCOS, underscoring the need for future functional and longitudinal studies to validate these genetic effects and elucidate the underlying mechanisms. While CYP11A1 variants may contribute to PCOS susceptibility in Bangladeshi women, the in-silico GTEx eQTL analysis did not show statistically significant effects on CYP11A1 gene expression in ovarian tissue. Therefore, functional consequences of these polymorphisms remain speculative. However, future research should focus on different ethnic groups, population-based control groups, and large-scale case–control studies to increase statistical power and confirm these genetic associations. Additionally, further laboratory-based assays and larger tissue-specific transcriptomic datasets will be needed to clarify the functional and structural implications of these SNPs on CYP11A1 gene expression.

## Supplementary Information

Below is the link to the electronic supplementary material.

Supplementary Material 1

## Data Availability

All data generated or analyzed during this study are included in this published article and its supplementary information files. Additional datasets used and/or analyzed during the current study are available from the corresponding author on reasonable request.
