# Supplementary material for: Genetic exploration of the impact of CYP11A1 polymorphisms on the development of PCOS: evidence from a meta-analysis with trial sequential analysis
Source: Sci Rep. 2026 Jun 3;16:25368. doi: 10.1038/s41598-026-50710-z (PMC13473682; doi:10.1038/s41598-026-50710-z)
Supplement: Supplementary file 1 [file 41598_2026_50710_MOESM1_ESM.docx]

**Table S1: Quality assessment of the included case-control studies by NOS**

| Study ID | Selection | | | |  | Comparability | |  | Exposure | | | Total Score |
| --- | --- | --- | --- | --- | --- | --- | --- | --- | --- | --- | --- | --- |
|  | 1 | 2 | 3 | 4 |  | 5A | 5B |  | 6 | 7 | 8 |  |
|  | Adequate Case Definition (★) | Representativeness of Cases (★) | Selection of Controls (★) | Definition of Controls (★) |  | Case-control adjusted for age | Case-control adjusted for other important factor(s) |  | Ascertainment of Exposure (★) | Same Method of Ascertainment (★) | Non-response Rate (★) |  |
| Gao et al 2010 | * | * | * | * |  | * | - |  | * | * | - | 7 |
| Zhang et al 2012 | * | * | - | * |  | - | - |  | * | * | - | 5 |
| Chen et al 2016 | * | * | * | * |  | * | - |  | * | * | - | 7 |
| Abdel-mageed et al 2016 | * | * | * | * |  | - | - |  | * | * | - | 6 |
| Shan et al 2016 | * | * | * | * |  | * | * |  | * | * | - | 8 |
| Kaur et al 2021 | * | * | * | * |  | * | * |  | * | * | - | 8 |
| Fathy et al 2023 | * | * | * | * |  | * | - |  | * | * | - | 7 |
| Sajid et al 2024 | * | * | * | * |  | * | - |  | * | * | - | 7 |
| Bashir et al 2025 | * | * | * | * |  | - | - |  | * | * | - | 6 |
| Rao et al 2025 | * | - | * | - |  | - | - |  | * | * | - | 4 |
| Aboelros et al 2025 | * | * | * | * |  | * | - |  | * | * | - | 7 |
| Wazir et al 2025 | * | * | * | * |  | * | - |  | * | * | - | 7 |

NOS=Newcastle Ottawa Scale; in this scale, the score ranges between 0-9, where “0-3” indicates low quality, “4-6” indicates moderate quality, and a score of ≥7 indicates a high quality study
